# Multi-isotopic and morphometric evidence for the migration of farmers leading up to the Inka conquest of the southern Andes

**DOI:** 10.1038/s41598-020-78013-x

**Published:** 2020-12-03

**Authors:** Ramiro Barberena, Lumila Menéndez, Petrus J. le Roux, Erik J. Marsh, Augusto Tessone, Paula Novellino, Gustavo Lucero, Julie Luyt, Judith Sealy, Marcelo Cardillo, Alejandra Gasco, Carina Llano, Cecilia Frigolé, Daniela Guevara, Gabriela Da Peña, Diego Winocur, Anahí Benítez, Luis Cornejo, Fernanda Falabella, César Méndez, Amalia Nuevo-Delaunay, Lorena Sanhueza, Francisca Santana Sagredo, Andrés Troncoso, Sol Zárate, Víctor A. Durán, Valeria Cortegoso

**Affiliations:** 1 Laboratorio de Paleoecología Humana, Instituto Interdisciplinario de Ciencias Básicas (ICB), Consejo Nacional de Investigaciones Científicas y Técnicas (CONICET), Facultad de Ciencias Exactas y Naturales, Universidad Nacional de Cuyo, Mendoza, Argentina; 2 Facultad de Filosofía y Letras, Universidad Nacional de Cuyo, Mendoza, Argentina; 3 Department of Anthropology of the Americas, University of Bonn, Bonn, Germany; 4 Konrad Lorenz Institute for Evolution and Cognition Research, Klosterneuburg, Austria; 5 Department of Geological Sciences, University of Cape Town, Cape Town, South Africa; 6 Instituto de Geocronología y Geología Isotópica, Universidad de Buenos Aires, Consejo Nacional de Investigaciones Científicas y Técnicas (CONICET), Ciudad Autónoma de Buenos Aires, Argentina; 7 Consejo Nacional de Investigaciones Científicas y Técnicas (CONICET), Museo de Ciencias Naturales y Antropológicas Juan C. Moyano, Mendoza, Argentina; 8 Departamento de Antropología, Facultad de Ciencias Sociales y Humanidades, Universidad Católica de Temuco, Temuco, Chile; 9 Archaeology Department, University of Cape Town, Cape Town, South Africa; 10 Instituto Multidisciplinario de Historia y Ciencias Humanas, Consejo Nacional de Investigaciones Científicas y Técnicas (CONICET), Ciudad Autónoma de Buenos Aires, Argentina; 11 Facultad de Ciencias Aplicadas a la Industria, Universidad Nacional de Cuyo, Mendoza, Argentina; 12 Departamento de Ciencias Geológicas, Instituto de Estudios Andinos (IDEAN), Universidad de Buenos Aires, Facultad de Ciencias Exactas y Naturales, Buenos Aires, Argentina; 13 Departamento de Antropología, Universidad Alberto Hurtado, Santiago, Chile; 14 Departamento de Antropología, Universidad de Chile, Santiago, Chile; 15 Centro de Investigación en Ecosistemas de la Patagonia, Coyhaique, Chile; 16 Pontificia Universidad Católica de Chile, Santiago, Chile

**Keywords:** Biogeochemistry, Environmental social sciences

## Abstract

We present isotopic and morphometric evidence suggesting the migration of farmers in the southern Andes in the period AD 1270–1420, leading up to the Inka conquest occurring ~ AD 1400. This is based on the interdisciplinary study of human remains from archaeological cemeteries in the Andean Uspallata Valley (Argentina), located in the southern frontier of the Inka Empire. The studied samples span AD 800–1500, encompassing the highly dynamic Late Intermediate Period and culminating with the imperial expansion. Our research combines a macro-regional study of human paleomobility and migration based on a new strontium isoscape across the Andes that allows identifying locals and migrants, a geometric morphometric analysis of cranio-facial morphology suggesting separate ancestral lineages, and a paleodietary reconstruction based on stable isotopes showing that the migrants had diets exceptionally high in C_4_ plants and largely based on maize agriculture. Significantly, this migration influx occurred during a period of regional demographic increase and would have been part of a widespread period of change in settlement patterns and population movements that preceded the Inka expansion. These processes increased local social diversity and may have been subsequently utilized by the Inka to channel interaction with the local societies.

## Introduction

Migrations are an intrinsic aspect of human societies in the present as in the past^1–3^. While their dynamics differ^4,5^, migrations occurred across levels of socio-economic complexity, from small-scale mobile societies to ancient states^6–10^. Archaeologists have long debated the role of migration and diffusion in stimulating change in past human societies. During the early twentieth century, migration was frequently invoked as the major driver of cultural change, but subsequently fell into disfavor^1,2,11^. Today, the application of radiogenic and stable isotopes enable us to distinguish migrants with confidence^12,13^, and together with the phylogenetical information provided by paleogenomics^14,15^ are bringing migration back to the forefront. However, current archaeological approaches concede migration a negligible role to explain economic and socio-demographic changes in the periphery of the ‘Andean’ world (although see^16,17^). With the aim of bridging the continental and regional scales, we develop a fine-grained study of human migration at local and regional scales in the southern Andes of Argentina and Chile. We argue here that it is essential to systematically study migration as a historical process in order to understand social and economic change in a more nuanced manner.

**Figure 1:**
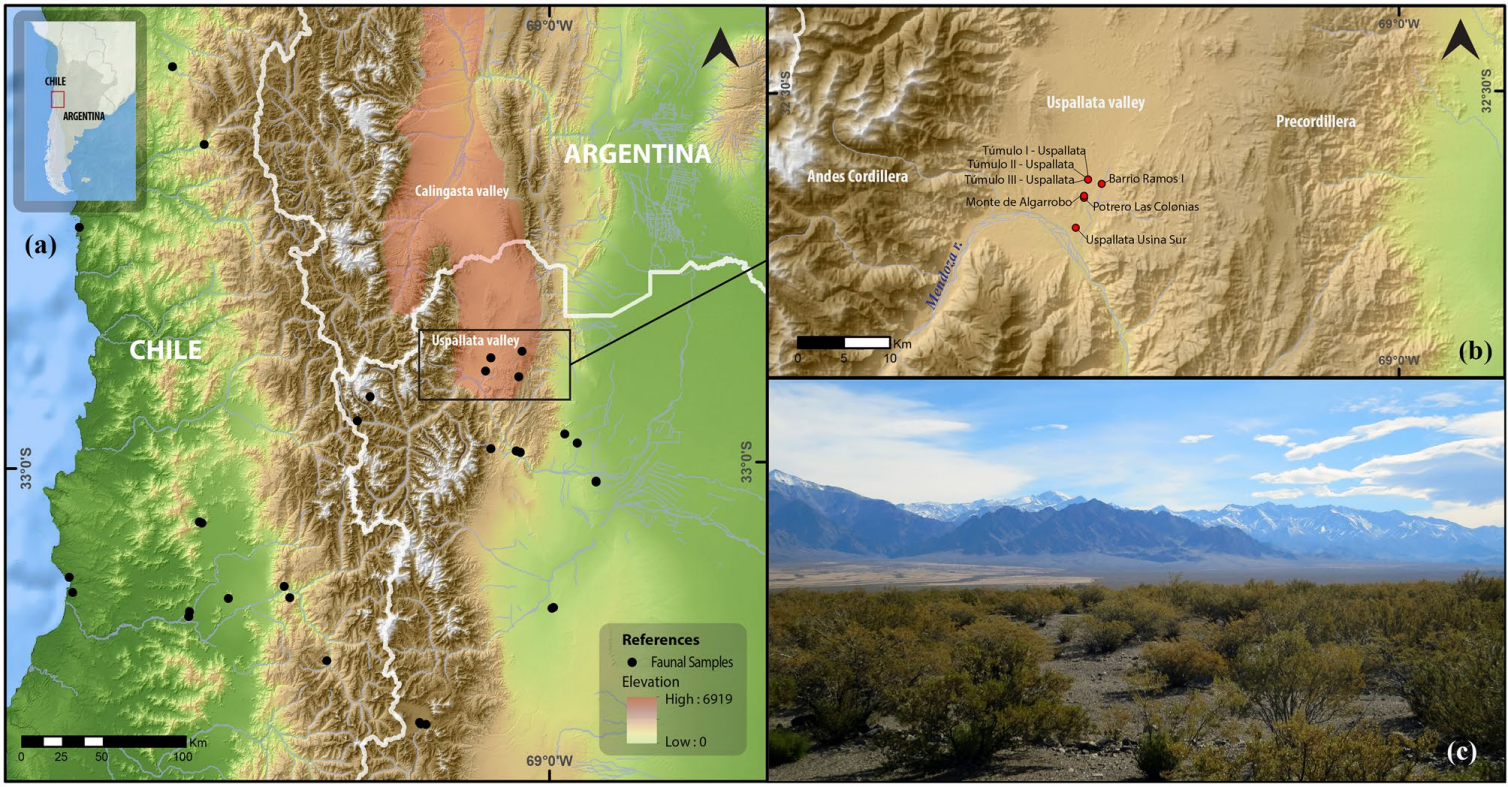
(**a**) Map of the southern Andes showing the study area (within the rectangle) and the locations of rodent sampling sites for bioavailable strontium (black dots); (**b**) Archaeological sites with human remains in the Uspallata Valley; (**c**) Panoramic view of the Uspallata Valley with the Andean Frontal Cordillera behind. Maps generated with Quantum GIS, version 3.2.3 (https://www.qgis.org) and edited with Inkscape 0.92 (https://inkscape.org).

This study is centered in the Uspallata Andean Valley in Mendoza Province, Argentina (Fig. 1). This region represents the southern frontier of the dispersion of Andean agropastoral economies with a southwards latitudinal pattern of decreasing agriculture importance between 32° and 34° S^17–20^. During the last ca. 2000 years, there is evidence of the use of domestic plant species such as maize (*Zea mays*), quinoa (*Chenopodium* spp.), beans (*Phaseolus vulgaris*), and squash (*Cucurbita* spp.), in addition to camelid herding (*Lama glama*) and hunting and gathering of wild animal and plant resources^18,21,22^. Recent studies of stable isotopes in human remains suggest mixed diets that do not reach the levels of maize consumption of dedicated agriculturalists between AD 800 and 1450, where variation in maize farming would have been strongly linked to fluctuations in temperature^21^. It has been suggested that peaks in δ^13^C_apatite_ values for humans during the interval AD 1250–1370 are consistent with positive anomalies in summer temperature^21^, hence linking trends in the intensification of agricultural practices to internal adaptive responses to climate change.

After AD 1400 Uspallata was the southernmost Inka administrative center east of the Andes, as shown by the presence of several archaeological sites with Inka-related architecture such as Ranchillos and Tambillos, and by its relationship with the Inka road or *Qhapaq Ñan*^23,24^. It is also close and geographically connected via the natural circulation path of the Mendoza River to the Aconcagua mummified child offering, which is associated to the Capacocha propitiatory ceremony^25,26^. Since the Uspallata Valley has a remarkable mortuary record spanning the last 1200 years^27–30^, it offers a unique window for studying the transition to productive economies, migration, and social interaction between diversely organized societies ranging from incipient farmers to expansive empires during a highly dynamic period along the Andes. Building from this regional case, we zoom-out to a macro-regional level to assess the broader historical context underlying these local processes. Our research focusses on the analysis of human life histories by studying place of residence and migration by means of strontium isotopes (^87^Sr/^86^Sr), dietary changes and the importance of maize agriculture based on stable isotopes of carbon and nitrogen, and phenotypic variation by means of cranio-facial 3D geometric morphometrics in human remains. Kernel Density Estimation (KDE) of radiocarbon dates and Bayesian statistics are combined to situate the geographic processes within regional trends of occupational intensity. The results of our study have implications for understanding economic shifts, demographic fluctuations, and multi-ethnic dynamics of interaction before and during the Inka expansion.

### Mortuary remains in an Andean biogeographic corridor

Uspallata is an intermontane valley located in northwestern Mendoza between two morpho-structural geological units, the Cordillera Frontal and Precordillera (Fig. 1). The valley is connected to other longitudinal valleys to the north, such as Calingasta, comprising a ~ 400 km-long biogeographic corridor^31^. Situated at an altitude of ~ 1900–2200 masl, the valley can be occupied year-round, unlike the higher areas surrounding it, where the winter snow-cover limits human occupation to the summer season. Hence, this valley is a key region for tracking human migrations across and along the southern Andes.

Near 200 individuals were buried between AD 800–1500 at seven clustered archaeological sites excavated in the first half of the twentieth century, including the cemetery sites Potrero Las Colonias (MNI = 119), Túmulo I (MNI = 29) and Túmulo III (MNI = 27) (Fig. 1, see archaeological contexts in Table 1)^28^.

**Table 1:** Archaeological contexts and isotopic sampling for the Uspallata Valley.

Site	Archaeological context	^14^C dates (BP)	95% probability (cal AD)	MNI	n ^87^Sr/^86^Sr	n Paleodiet (+)	Age classes		Sex			References
							SA	A	M	F	ND	
Túmulo I	Cemetery with primary burials	977 ± 35 (AA-66568)	1020–1190	29	4	8	7	22	7	7	15	^21, 28^
Túmulo II	Cemetery with primary burials and diverse associated grave goods	1178 ± 41 (AA-66565)	770–1020	10	14	15	3	7	3	3	4	^28, 32^
		1269 ± 35 (AA-66561)	680–890									
Túmulo III	Cemetery with primary burials	671 ± 40 (AA-66566)	1290–1400	27	3	8	14	13	1	4	22	^21, 28^
Potrero Las Colonias	Ossuary with no associated grave goods	682 ± 25 (D-AMS-033194)*	1290–1400	119	7	10	54	65	22	25	72	^21, 28^
		634 ± 28 (D-AMS-031415)*	1300–1420									
		568 ± 38 (AA-66564)	1320–1450									
Barrio Ramos I	Multiple individuals, Inka-period grave goods	583 ± 43 (AA-98708)	1310–1450	6	6	3	3	3	2	1	3	^30^
		470 ± 80 (I-16636)	1320–1650									
		AD 1400 ± 60 (UCTL-308)	1280–1520									
Usina Sur 2	Primary burial, no grave goods	772 ± 25 (D-AMS-033193)*	1220–1380	4	2	2	2	2	0	1	3	This paper
Monte de Algarrobos	One individual, primary burial with grave goods	298 ± 28 (D-AMS-030192)*	1500–1800	1	2	2	0	1	0	1	0	^28^, this paper
Total				196	38	48	83	113	35	42	119	

Key: (*) date reported here for the first time; (+) includes new and previously published results. All dates recalibrated with SHCal20^101^.

## Results

### Bioavailable strontium, local residence, and immigration: a diachronic assessment

**Figure 2:**
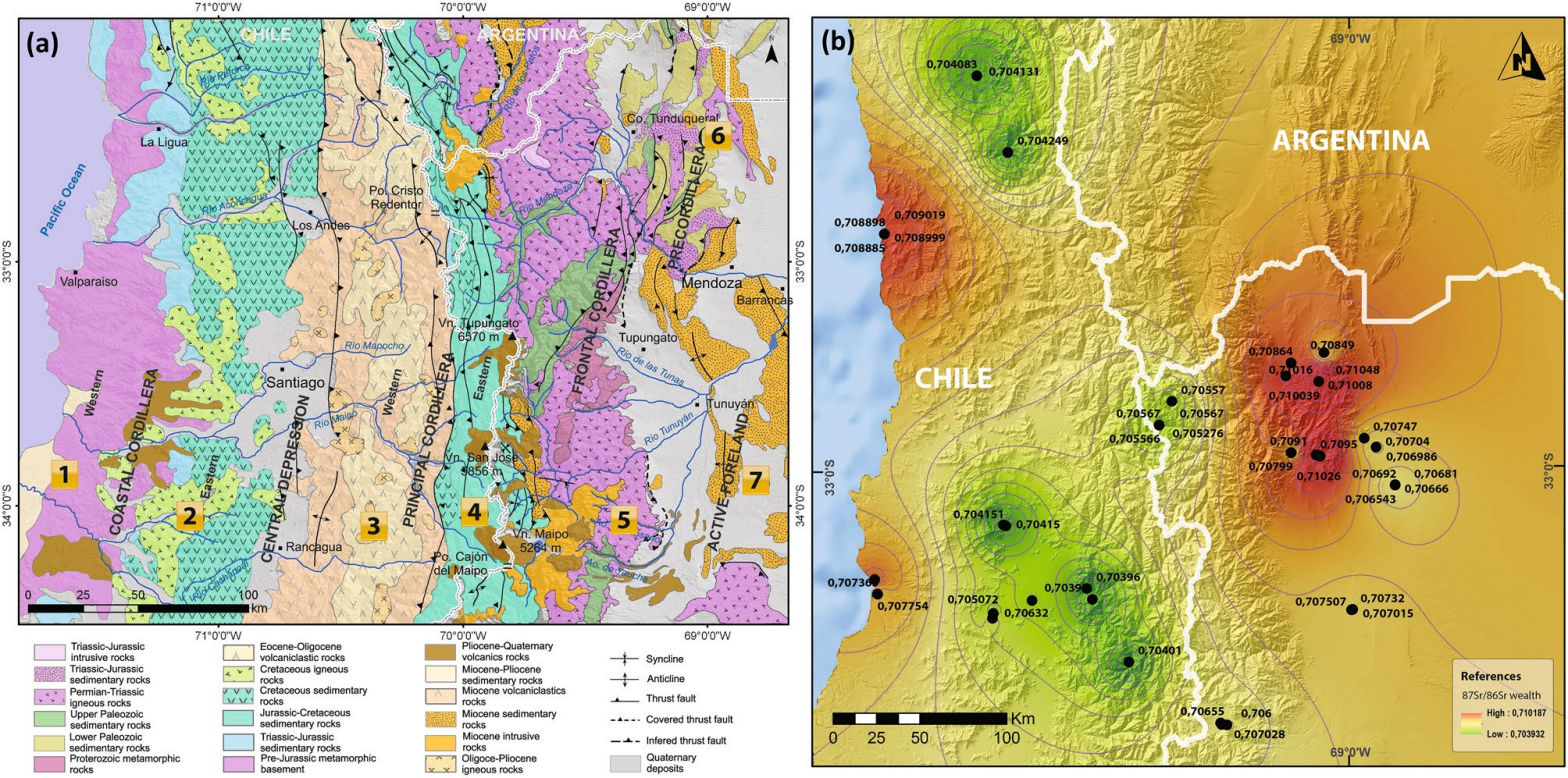
Framework for the study of bioavailable strontium in the southern Andes: (**a**) Main geological provinces: (1) Pacific Coast, (2) Coastal Cordillera, (3) Eastern Principal Cordillera, (4) Western Principal Cordillera, (5) Frontal Cordillera; (6) Precordillera, (7) Quaternary Active Foreland; (**b**) Isoscape of strontium isotopes (^87^Sr/^86^Sr) in rodent samples for the southern Andes of central Argentina and Chile. Map generated with Quantum GIS, version 3.2.3 (https://www.qgis.org) and edited with Inkscape 0.92 (https://inkscape.org). (**a**) Using public domain data from SEGEMAR (https://sigam.segemar.gov.ar/visor/) and modified from^33^ (Copyright Clearance Center License ID 1055264-1).

The geology of the southern Andes is especially suited for tracking local residence and immigration to the Uspallata Valley due to the high diversity of bedrock age and composition in a restricted area (Fig. 2a; see^33,34^). Biologically available strontium from each geological unit was characterized by the analysis of modern and archaeological rodent samples with restricted home ranges, which are appropriate for developing a baseline to compare to human samples^35–37^. We analyzed 65 samples collected from the main geological units along a 250-km transect from the Pacific coast in Chile to the lowlands in the eastern Andean slope (Table S1). The results show that the rodent samples closely track the geological regions. There is minimal isotopic overlap between areas of key archaeological significance such as western Principal Cordillera, in Central Chile, eastern Principal and Frontal Cordillera—making up the highlands—and Precordillera—east of the Andes—(Fig. S1). The Uspallata Valley is characterized by highly radiogenic values derived from the Paleozoic Precordillera along its eastern flank, composed of the oldest Andean formations (~ 500–350 my^38,39^), that transfer a distinct isotopic signature to the valley (Fig. 2B). While our results show similar values for the coastal environments of the Pacific Ocean, partially incorporating marine strontium^40^, a multi-isotopic approach combining paleodietary tracers allows assessing the dietary source of strontium^41,42^ and accurately tracking local and non-local residents in Uspallata.

**Figure 3:**
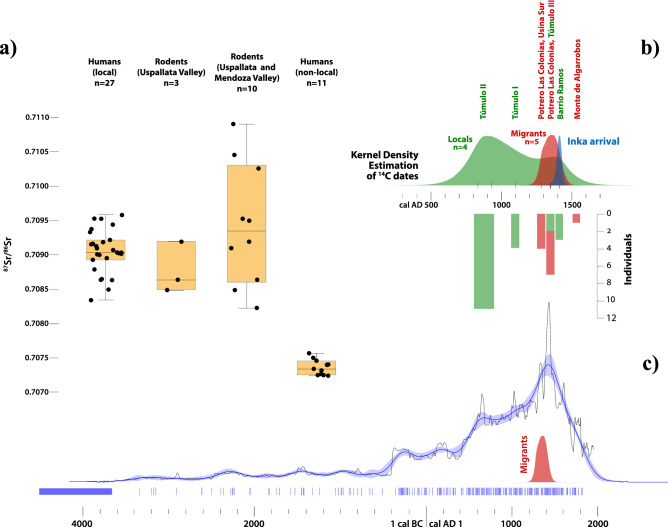
(**a**) Strontium values from human and rodent samples from the Uspallata Valley and nearby areas; (**b**) Bayesian modelling, KDE, and inverted histogram of individuals with local (green) and non-local (red) values compared to the estimated date (blue) for the Inka arrival; (**c**) Summed probabilities (thin black line) and KDE (blue curve) for radiocarbon dates from Mendoza^43^ compared to the KDE for migrants. Figures generated with Excel 16.39 (ID: 02954-035-637535) and OxCal 4.3 (https://c14.arch.ox.ac.uk/oxcal.html) and edited with Inkscape 0.92 (https://inkscape.org).

We present ^87^Sr/^86^Sr values for 38 human samples (12 teeth and 26 bones) from 30 individuals spanning the period between AD 800 and 1500, representing 15% of the human remains from these burial sites (MNI = 196) (Table S2). The results have a bimodal distribution with two non-overlapping groups: the first one is composed of 27 samples (11 teeth and 16 bones) with a mean of 0.7090 ± 0.0003 (range 0.7083–0.7095); the second group is composed of 11 samples (one tooth and 10 bones) with a mean of 0.7073 ± 0.0001 (range 0.7072–0.7075) (Fig. 3a). The distribution of the values in the sample is multimodal (Hartigans’ dip test, *D* = 0.10455, *p* = 0.001) and the two groups present statistically significant differences (Mann–Whitney *z* = 4.7637, *p* =  < 0.0001).

The first group (n = 27) overlaps with the isotopic range defined by ten rodent samples from Uspallata and the adjacent Mendoza River valley (Fig. 3a), defining a local isotopic range for the area of contact between Frontal Cordillera and Precordillera that encompasses the Uspallata Valley. Within this area, the more radiogenic rodent samples come from the Mendoza River, which receives more sediment from the Paleozoic Precordillera (Fig. S1). The second group of human samples (n = 11) has a restricted isotopic variation that does not overlap with the local baseline (Fig. 3a).

Based on our isoscape, we assign probable areas of residence for these two groups with focal statistics based on each group’s mean and two-standard deviation range (Fig. S2). The 27 values from the first group (^87^Sr/^86^Sr range = 0.7087–0.7096) fully coincide with the local baseline. While the geographical analysis also indicates the Pacific coast as a possible area of residence, the paleodietary results—presented below—show a null marine dietary input, therefore allowing to reject this alternative^21^. Hence, we argue that these samples indicate residence in or near the Uspallata Valley during childhood and/or adulthood. The spatial analysis also shows that the second group of 11 individuals (^87^Sr/^86^Sr range = 0.7071–0.7075) did not reside in Uspallata or nearby areas and are most likely migrants. However, the results are not conclusive regarding their geographic origin. Two distinct possibilities are the lowlands to the southeast and the highlands to the north (Fig. S2). However, since isotopic equifinality has been recorded in multiple Andean settings^37^, they may also be long-distance migrants from beyond the sampled area.

There is a patterned temporal trend in the distribution of these isotopic groups (Fig. 3b). The 27 local samples come from four sites spanning AD 800–1500, which indicates the continuous presence of locals in the Valley until the Inka arrival ~ AD 1410, as estimated by a regional Bayesian model^44^. The three earliest dates from the sites Túmulo I and Túmulo II (AD 830, 930, and 1100) are directly associated with 19 samples from 15 individuals with local ^87^Sr/^86^Sr values. On the other hand, the non-local individuals are temporally clustered between ~ AD 1280–1420 (*First* and *Last* medians), a brief phase that includes 10 of the 11 non-locals from the sites Potrero Las Colonias, Usina Sur 2, and Túmulo III.

The mortuary assemblages provide some cultural context for the local individuals from the early and late periods of the sequence (Table 1). Three individuals buried at Túmulo II (AD 800–1000) are respectively associated with a lithic lip plug—individual 239—a lithic projectile point—individual 241—and two complete ceramic vessels assigned to pottery traditions from the penecontemporaneous Early Ceramic Period in Central Chile^18,32^—individual 245—(Fig. S3). These vessels probably arrived via exchange and were then incorporated in the burials, suggesting they carried social and ritual significance. Furthermore, three individuals from Barrio Ramos I site (AD 1420), for whom paired bone and teeth ^87^Sr/^86^Sr values establish local residence throughout life, are associated with hundreds of shell beads—*Dyplodon chilensis*—a lithic projectile point, and large bone projectile points generally associated to the Inka period (Fig. S3)^30,44^. Most of these cultural elements are consistent with the local residence as determined isotopically.

Conversely, the sites from which the migrants come do not contain material elements that may shed light on their cultural affiliation. The sites Potrero Las Colonias and Túmulo III are ossuaries^28^ with large numbers of individuals deposited in restricted pits, without any associated grave goods and with a high representation of subadults. Four direct dates from these sites have modeled medians of AD 1350, 1350, 1350, and 1410. Potrero Las Colonias site contains the remains of a minimum number of 119 individuals, including 54 subadults and 65 adults. We randomly selected seven individuals from this site and all seven had non-local strontium values. Túmulo III yielded a minimum number of 27 individuals, composed of 14 subadults and 13 adults (Table 1). One of the three individuals analyzed from this site produced a non-local signature and the other two were determined as local. These results suggest that locals and migrants lived—and in some cases were buried—side by side.

Zooming out from these regional patterns, the timing of the immigrants’ arrival coincides with the onset of the major regional occupational peak, as suggested by a KDE of radiocarbon dates from Mendoza Province (n = 343; Fig. 3c)^43^. While the temporal trends cannot be treated as a direct demographic proxy, it is notable that the long-term occupational peak coincides with the formation of large cemeteries with several subadults^45,46^, possibly indicating a macro-regional demographic increase coeval to the period of the migration(s).

### Cranio-facial morphology and geographic origin

**Figure 4:**
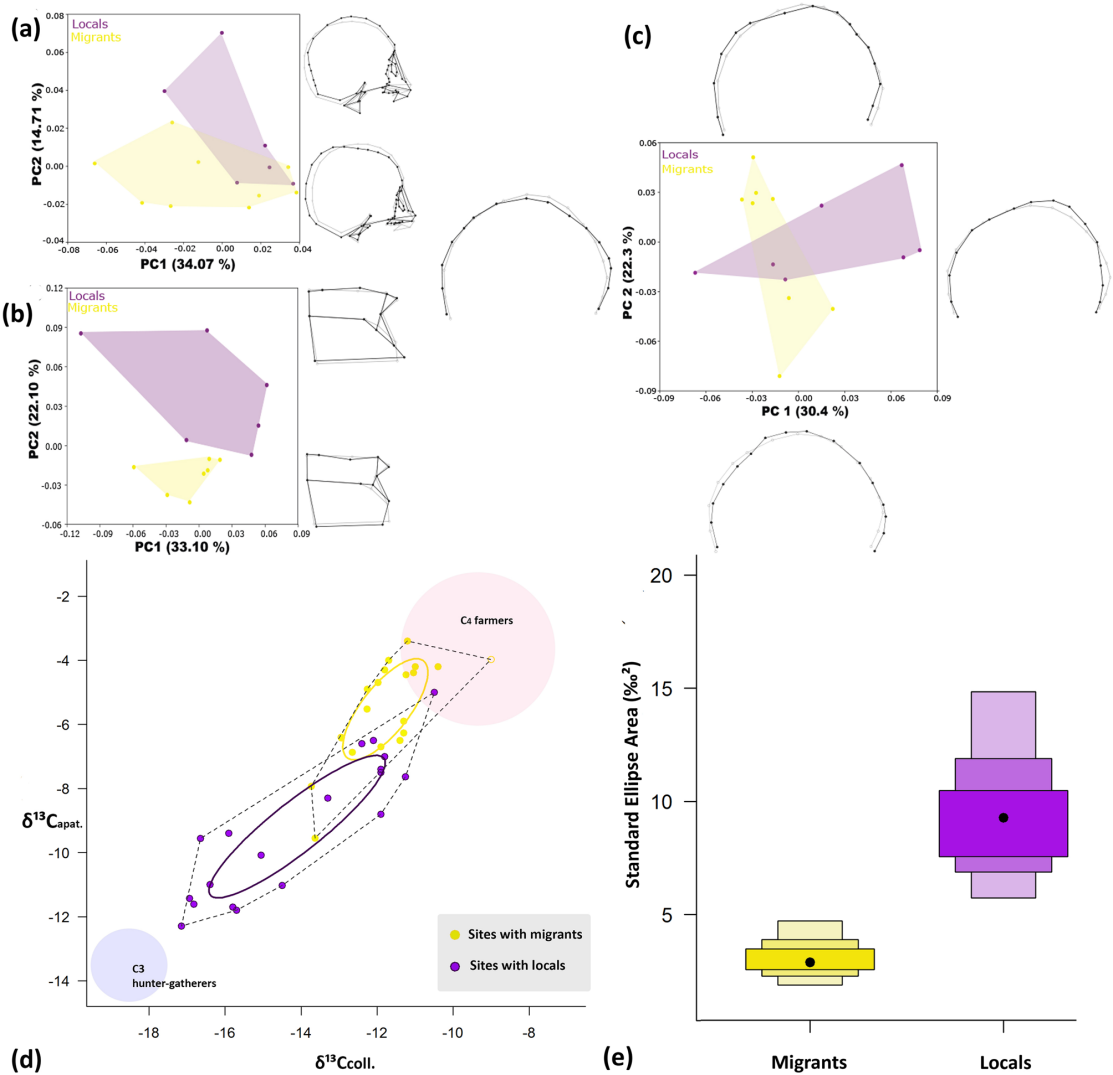
(**a**) Cranio-facial geometric variation and paleodiet in human remains from Uspallata: PCA of cranio-facial variation; (**b**) PCA of the cranial base variation; (**c**) PCA of the cranial vault variation; (**d**) δ^13^C_collagen_ and δ^13^C_apatite_ in sites from Uspallata compared to theoretical endmembers for C_3_ hunter-gatherer and C_4_ farmer diets^50,51^, total area of the group—dotted line—and Standard Ellipse Area corrected for small sample size (SEA_C_)—continuous line; and (**e**) Standard Ellipse Area Bayesian (SEA_B_). Figures generated with R version 3.6.2 (https://www.R-project.org) and Excel 16.39 (ID: 02954-035-637535) and edited with Inkscape 0.92 (https://inkscape.org).

Cranial morphology confirms the bimodal arrangement between locals and non-locals. While the first two principal components explain 50% of the entire skull shape variation, there is some overlap among local and non-local individuals (Fig. 4a). The main variables that contribute to such differences are landmarks and semilandmarks located along the sagittal plane of the cranial vault (Table S3). Since previous studies have shown that the morphological variation in these samples is low due to recent divergence^17, 47^, we conducted further analyses by using only the cranial base. The development of this cranial module is completed earlier during ontogeny, making it less determined by environmental conditions and thus retaining a stronger population history record^48,49^.

The first two principal components of the cranial base explain more than 55% of the total variation and reveal strong differences between the local individuals buried at Túmulo II (n = 6) and the non-locals buried at Potrero Las Colonias (n = 7; Fig. 4b). Most of the variation is concentrated in the temporal and occipital bones—anterior and inferior mastoid, asterion, and basion—(Table S4). This trend is reinforced by the results of a Procrustes ANOVA that showed significant statistical differences between cranial base shapes (F = 1.83; *p* ≤ 0.01). This is not the case when comparing the whole skull, despite the low p value (F = 1.18; *p* = 0.06). The observation that the largest differences between locals and non-locals are present in the cranial base supports the conclusion that these groups have different lineages, which nonetheless might have shared a common ancestor in the deep past^52^.

In order to test for differences in cultural modifications of the skull among the local and non-local individuals, we also evaluated shape variation along the sagittal curvature of the cranial vault. Previous research showed the presence of wide variation of artificial cranial modifications in the southern Andes, which could be interpreted as a potential manifestation of ethnic identity^53,54^. The first two principal components explain 52% of the total cranial vault shape variation (Fig. 4c). The results from the PCA show that the cranial vault of the locals varies across PC1, with some of individuals presenting a flattening in the occipital area, while others do not present cranial modifications (Table S5). The non-locals’ cranial vault has a wider range of cultural modifications that goes across the PC2, from a subtle flattening of the frontal bone to a flattening of the lambdic area (Table S5). Procrustes ANOVA showed that there are some statistically significant differences between the locals and non-locals cranial vault shape (F = 1.90; *p* ≤ 0.01). Despite the partial overlap recorded for the cranial vault shape variation of locals and non-locals, the differential pattern of cultural cranial modifications could support the existence of identity differences among them. However, we cannot yet discard other factors.

### Paleodiets and maize agriculture

We present results of δ^13^C_col._, δ^13^C_apat._, and δ^15^N for 22 individuals (6 teeth and 19 bone samples), which are combined with previously published values for the same sites^21^. Since teeth samples are unevenly distributed between sites and most teeth represent diet during childhood, we focus the following analyses on the bone samples, which reflect a longer-term average of diet over many years of life. Thus, we have values for bone samples from 39 individuals distributed as follows: 20 from sites with migrants—Potrero Las Colonias, Túmulo III, Usina Sur 2, and Monte de Algarrobos—and 19 from sites with local individuals only—Túmulo I, Túmulo II, and Barrio Ramos I—(Table S6). Mann–Whitney pair-testing shows significant differences (*p* < 0.05) in δ^13^C_coll._ and δ^13^C_apat._, while the sites are not statistically different in terms of δ^15^N (Table S7).

The locals show more variability in the input of C_3_ and C_4_ resources than the migrants, as determined with both δ^13^C_coll._ and δ^13^C_apat._ (Fig. 4d; see also Fig. S4). This is visible in the total isotopic area occupied by each group. In addition, a Bayesian analysis of the metrics of the isotopic areas by means of the Standard Ellipse Area corrected for small sample size—SEA_C_—(Fig. 4d) produces values of 3.881 for the migrants and 7.377 for the locals (Table S8). The Standard Ellipse Area Bayesian—SEA_B_^55^ also shows a smaller isotopic niche for the migrants (3.224) compared to the locals (9.913; *p* = 1) (Fig. 4e; Table S8). The enriched ^13^C_apat._ values from the sites with migrants suggest a high C_4_ dietary input that, based on the regional isotopic ecology^21,56^, is compatible with an agricultural subsistence heavily reliant on maize cultivation (Fig. 4d). While alternative dietary factors for C_4_ dietary inputs have been suggested, such as CAM plants and enriched wild herbivores^57,58^, we do not consider these alternatives can account for the very restricted isotopic niches near the most enriched end of the distribution, as recorded for the migrants in this study. Similar values recorded for δ^15^N among migrants and locals would suggest an important protein intake from wild herbivores, such as the guanaco (*Lama guanicoe*). Importantly in this context, comprehensive studies for wild herbivores in Mendoza do not record δ^13^C values that could explain the particularly enriched migrant values^56^. In addition, a study of taxon-dates for macro-botanical remains of cultigens from Mendoza including maize identifies the highest abundance between ca. AD 1300–1500^43^.

## Discussion and conclusions

We have presented ^87^Sr/^86^Sr values from human remains that indicate a migration influx into the southern Andean Uspallata Valley occurring between AD 1280 and 1420. Stable isotopes suggest that a large fraction of the migrants came from farming communities that practiced maize agriculture, as opposed to a broader subsistence base recorded for the local groups that included maize in variable proportions alongside C_3_ plants and camelids. At a biological level, in addition to the bimodal arrangement of the cranio-facial variation (Fig. 4a), there is a significant difference in the shape of the cranial base (Fig. 4b), which preserves a strong signature of the population lineage. Additionally, the observed shape variation in the cranial vault would suggest cultural patterns of cranial modifications possibly associated with ethnic identity^59,60^, where the migrants present a wider range of cranial modifications in the frontal and occipital bones (Fig. 4c), despite their remarkable dietary and geographic homogeneity. These lines of evidence suggest that, in the period between AD 800 and 1500, locals and migrants can be distinguished on the basis of geographic origin, phenotypic features, cultural body modifications, dietary breadth, and possibly agricultural practices.

We have provided evidence for the coexistence of human groups with biological and economic differences in Uspallata, but we know little about the dynamics of their interaction. Remarkably, the migrants buried in cemeteries such as Potrero Las Colonias and Túmulo III retained a homogeneous non-local signature in their bones. An ongoing analysis of these remains does not reveal clear signs of violence as the cause of death, but the presence of diseases still needs to be explored by applying an interdisciplinary framework^61^. While migrants’ origin has not been confidently determined yet, we can rule out on isotopic grounds the possibility that they came from the highly populated Chilean Central Valley. There is, among others, one plausible alternative along the biogeographic corridor of intermontane valleys to the north. Indeed, a contemporary emigration from the Angualasto area, emplaced in the same biogeographic corridor 250 km northwards from Uspallata, has been suggested^16,62^, as well as similarities between the Angualasto ceramic tradition and the local Viluco style from northern Mendoza^63^. In addition, while there is contextual cultural information on the locals throughout the temporal sequence consistent with the isotopic results (Fig. S3), the mortuary contexts of the migrants do not contain associated grave goods that may inform on their cultural identity. The social context accounting for the complete absence of cultural materials on the large cemeteries containing the migrant burials is unknown and will be explored in the future.

Most of the migrants had homogeneous diets that occupy very restricted isotopic niches and were focused on the consumption of C_4_ foods, most likely maize. Overall, despite the fact that sample size needs to be enlarged, the remarkable geographic and dietary homogeneity reconstructed for the migrants, coupled with the emphasis on maize agriculture and the large numbers of individuals buried, suggest a picture of low mobility, very stable life-histories, and possibly high demography in the source area^64,65^, which provides a plausible ‘push’ context for population displacement. In this regard, it must be emphasized that the δ^13^C_apat._ values are higher than those recorded for most of the regional records known in central Argentina and Chile^21,66,67^, and almost as enriched as cases from the Quebrada de Humahuaca in northwestern Argentina, northern Chile, and Conchopata in central Peru^68–70^ (Fig. S5).

Current models of the development of agriculture in this region are largely based on isotopic information^21,43^. This framework has emphasized internal processes of economic adjustment in response to climate change, with maize consumption peaking between AD 1250 and 1370 during a period of positive anomalies in summer temperatures and then declining during the Little Ice Age^21^. Our results suggest that the peak in the intensification of maize farming is largely evidenced in samples from archaeological sites constituted by migrants (Table S6). Whilst this new pattern does not necessarily contradict the long-term influence of climate change on agricultural intensification, it demonstrates the need to build more sophisticated models that incorporate migration in addition to in situ adaptive change^21,71^ to understand the complex processes of economic and demographic transition in the southern Andes. As it has already been explored^72^, this complex and intensively studied diachronic trajectory from the southern Andean farming frontier needs to be fully incorporated in comparative studies of transitions to productive economies^73–75^.

The immigration recorded in this paper shortly precedes the initial presence of the Inka in Uspallata, leading us to suggest that there was a multicultural social setting when the Empire arrived after ~ AD 1400. This entails a more complex dynamic of interaction between the Inka and the diverse preexisting local societies in the southern periphery, where the Inka may have established different forms of ‘top-down’ interaction with particular local social segments, as shown for other areas in the Tawantinsuyu^76–80^, and local groups may also have pursued their own agendas, as recent ‘bottom-up’ perspectives suggest^77,79,80^. Importantly for future research, frontier regions such as Uspallata should be considered as highly dynamic spaces combining evolving identities^77^.

Finally, our results also have macro-regional implications. The period between AD 1280 and 1420 is associated with changes at different spatial scales along the Andes. Regionally, it coincides with the major demographic peak in central Argentina and Chile. At a larger scale encompassing Peru to northern Chile and northwestern Argentina, this period of time—traditionally referred as the Regional Developments or Late Intermediate Period—is characterized by marked changes in settlement patterns, an increased development of regional identities, economic intensification, and more intense inter-ethnic conflict^18,70,81–84^ in a time of recurrent and intense dry periods^85^. While there is no firm evidence of conflict in the study area during this period, we should explore the possibility that these disparate trajectories may actually represent local manifestations of large-scale trajectories of demographic growth, enhanced push–pull dynamics^1^, and social change. Summarizing the results from the multiple lines of research developed here, we have presented compelling interdisciplinary evidence for the existence of a migration pulse of maize-farming groups shortly preceding the Inka conquest towards the imperial periphery. In doing so, we have contributed to open new avenues for research in the southern Andes by building a multi-scalar approach to human life histories^86^. We look forward to combining work at a micro-social level by reconstructing individual life-histories of migrants and locals, including possible causes of death such as violence or diseases, and at a macro-biological level, by contributing to reconstruct the paleogenomic history^15,52^ of these diverse societies of the Andes.

## Materials and methods

### Samples

The archaeological samples studied are from human remains deposited at the *Museo de Ciencias Naturales y Antropológicas Juan Cornelio Moyano* (Mendoza, Argentina). Sex and age determinations for paleodemographic analysis were performed according to standard procedures^87^. The applied methods are detailed in the “Supplementary Information”. The category subadult includes individuals ranging from prenatal stages to 20–21 years, and was defined based on dental development and the fusion of epiphysis and diaphysis in the postcranial skeleton^87,88^. Individuals were considered adults (older than 21 years old) when the spheno-occipital synchondrosis is closed, the molar 3 is in eruption, and/or epiphysis and diaphysis of the postcranial skeleton are fused^87,89^.

### Radiogenic isotope analysis

Strontium isotopes (^87^Sr/^86^Sr) in the landscape vary according to bedrock age and composition and can be used to determine the geographic sources of dietary strontium in human tissues, and hence the scale of mobility during the period of tissue formation^90–92^. The ^87^Sr/^86^Sr analyses were performed at the Department of Geological Sciences, University of Cape Town, South Africa. Powdered samples were processed following routine chemical and MC–ICP–MS methods^90^. ^87^Sr/^86^Sr data are referenced to a value of 0.710255 for the international standard SRM987. Repeated analyses of an in-house carbonate reference material NM95 were processed as unknown along with samples from this study, yielding an ^87^Sr/^86^Sr average (0.708912 ± 0.000037 2σ; n = 17) in agreement with long-term data from this facility (0.708911 ± 0.000040 2σ; n = 414 over > 8 years). The bioavailable Sr isoscape was produced with *QGIS* 3.10 and *ArcGIS* 10.5.GIS. ^87^Sr/^86^Sr values from rodent samples were interpolated with Ordinary Kriging^37,93^. The areas of human residence were determined with the tools Focal Statistics and Reclassify.

The possibility of diagenetic strontium uptake from the soil cannot be completely ruled out, particularly in the bone samples, but we consider that the patterns in the data are not the product of diagenesis. Firstly, we have conducted a pilot study with Ca/P and U/Ca elemental concentrations including some of the rodent and human samples from this paper. Ca/P values range between 1.9 and 2.1, within the range of values characteristic of modern hydroxyapatite^94,95^ and the results obtained for U/Ca are consistent with little diagenetic contamination^33^. This is consistent with general expectations for minimal diagenesis in recent samples from dry climates, like those presented here^96^. The migrant bone samples from Potrero Las Colonias, which would be the most susceptible to incorporating local strontium from the soil, produced an almost identical non-local signal. Other independent data sets indicate coherence in these geographical assignations, including radiogenic and stable isotopes and cranial variation.

### Stable isotope analysis

In order to extract the bone apatite for paleodietary analyses, bone fragments were ground in a liquid nitrogen-cooled SPEX mill and sieved through 180 and 106 μm mesh. Only the 179–107 μm fraction was analyzed^50^. Approximately 2 mg of each apatite powder were reacted with 100% phosphoric acid in a ThermoFinnigan model II gas bench and the resultant CO_2_ gas passed into a ThermoFinnigan Delta Plus XP isotope ratio mass spectrometer (Germany). Precision was monitored by repeated analysis of international standard materials (IAEA-CO1 and NBS18) in each run. ^13^C/^12^C and ^18^O/^16^O ratios were reported in the standard notation relative to the PeeDee Belemnite (PDB) standard in parts per mil (‰). Precision of repeated analyses of standard materials was < 0.2‰ for δ^13^C and δ^18^O. Bone collagen was prepared using the pseudomorph method described in^97^. Each sample was mechanically cleaned and weighed to enable determination of the collagen yield. The gases were passed through to a Delta Plus V IRMS (Thermo electron, Bremen, Germany), via a Conflo IV gas control unit (Thermo Finnigan, Bremen, Germany). Standards used were in-house standards Chocolate (δ^13^C = − 17.75‰; δ^15^N = 4.31‰); New MG (δ^13^C = − 20.94‰; δ^15^N = 4.7‰; Sucrose (δ^13^C =−10 .6‰); Valine (δ^13^C = − 26.80‰; δ^15^N = 12.14‰). Each standard had been calibrated against international standard materials NBS 21, IAEA N1 and N2 and standards exchanged with other laboratories. The reproducibility of repeated measurements of standard materials was ≤ 0.2‰.The human collagen samples showed good preservation with mean %N 15 ± 0.9, %C 41 ± 2.7 and C/N values between 3.0 and 3.4 (3.2 ± 0.1, Table S6)^98^. Only sample 31 presents an anomalous C/N value of 3.9 and was excluded from the analysis (Table S2). Only six of the previously published results do not include C/N ratios. Stable isotope results are analyzed with Stable Isotope Bayesian Ellipses (SIBER) in R^55,99^, using the Standard Ellipse Area corrected for small sample sizes (SEA_C_) and a Bayesian estimate of the Standard Ellipse Area (SEA_B_). The existence of significant differences in SEA_B_ between the migrants and locals were calculated on the basis of *p* values. A *p* value of 1 implies that the ellipse of the group with migrants is significantly smaller than the group with locals; conversely, a *p* value of 0 would imply that the ellipse of the local group is smaller and a *p* value of 0.5 means that the two populations have equal-sized ellipses^100^.

### Chronology

Radiocarbon dates were calibrated with SHCal20^101^ and modeled with OxCal 4.4^102^. We modeled two overlapping phases, one for dates from local burials and another for migrants. The dates provided in the text are medians rounded to the nearest decade for ease of presentation, however, error ranges are also considered. Modeled dates are in italics. A KDE for each phase is shown in Fig. 3B, in addition to an estimate of the initial Inka presence in the Uspallata Valley based on Bayesian models^103^.

### Craniometric variation

For the geometric morphometric analysis of cranio-facial variation, a total of 56 3D landmarks were registered by one of the authors (LM) by using a Microscribe G2X and taking into consideration intra-observer error (Table S9)^104^. Landmarks were selected for describing variation in the whole skull and were located sagittally on the left side of the skull. Intra-observer error was previously calculated for each landmark by comparing three independent measurement series, considered to be acceptable for morphometric studies (r = 0.90). Due to differential bone preservation, we selected 21 individuals to conduct the morphometric analysis (Table S10). The original coordinates were rotated, translated, and scaled with the Generalized Procrustes Superimposition method^105^. Afterwards, we obtained the Centroid size, a variable that designates size of the samples, and Procrustes coordinates that represent shape variables. Analyses were conducted in different cranial modules (whole skull, cranial base, cranial vault), which are expected to have different evolutionary and biocultural signals. Despite the fact that samples include approximately equal numbers of individuals of both sexes (Table S10), we performed a Procrustes ANOVA for evaluating the impact of sexual dimorphism in craniofacial shape variation. Since there are significant differences in shape variation due to sexual dimorphism (< 0.01), we run a regression of shape on size to obtain new shape variables uncorrelated with size in order to control for those sex-related differences. We used the residuals from the regression to perform a Principal Component Analysis that allowed reducing the number of variables by creating new orthogonal ones summarizing the variation. The variation along the first two principal components is presented with the wireframes that show the main shape configurations at the positive and negative extremes of the principal components compared (locals vs. non-locals). Mahalanobis distances were calculated to evaluate the magnitude of variation. Then, we conducted a Procrustes ANOVA to test the statistical significance of the differences between local and non-local groups. The analyses were conducted with MorphoJ and using the geomorph package (version 3.3.1)^106,107^ in R (version 3.6.2)^99^.

## Supplementary information

Supplementary Information.
