# Supplementary material for: Multi-isotopic and morphometric evidence for the migration of farmers leading up to the Inka conquest of the southern Andes
Source: Sci Rep. 2020 Dec 3;10:21171. doi: 10.1038/s41598-020-78013-x (PMC7712914; doi:10.1038/s41598-020-78013-x)
Supplement: Supplementary file 1 — Supplementary Information. [file 41598_2020_78013_MOESM1_ESM.pdf]

Supplementary Information

Multi-isotopic and morphometric evidence for the migration of farmers leading up to the Inka conquest of the southern Andes

Ramiro Barberena[1,2], Lumila Menéndez[3,4], Petrus J. le Roux[5], Erik J. Marsh[1,2], Augusto Tessone[6], Paula Novellino[7], Gustavo Lucero[8], Julie Luyt[9], Judith Sealy[9], Marcelo Cardillo[10], Alejandra Gasco[1,2], Carina Llano[1,11], Cecilia Frigolé[1,2], Daniela Guevara[7], Gabriela Da Peña[7], Diego Winocur[12], Anahí Benítez[12], Luis Cornejo[13], Fernanda Falabella[14], César Méndez[15], Amalia Nuevo-Delaunay[15], Lorena Sanhueza[14], Francisca Santana Sagredo[16], Andrés Troncoso[14], Sol Zárate[1], Víctor A. Durán[1,2] & Valeria Cortegoso[1,2]

* Correspondence to: rbarberena@mendoza-conicet.gob.ar; menendez@uni-bonn.de

Supplementary Text

Methods

Strontium isotopes: lab procedures and statistical analysis. The separation chemistry was performed in the clean-chemistry laboratory of the MC-ICP-MS facility in the Department of Geological Sciences, University of Cape Town (UCT)[1], using ultra-pure 2-bottle-distilled HNO_3. Powdered samples were weighed into 7 ml Savillex Teflon beakers, 2-3 ml of 65% HNO_3 added and the closed beakers placed at 140ºC for an hour. Following complete sample dissolution, the beakers were opened, and the samples dried. The samples were taken up in 1.5 ml of 2M HNO_3 in preparation for elemental separation chemistry. The Sr fraction was isolated after methods detailed in[2] using Triskem International Sr.Spec. resin, dried down and re-dissolved in 0.2% HNO_3. $^{87}Sr/^{86}Sr$ isotope analyses were performed using a Nu Instruments NuPlasma HR in the MC-ICP-MS facility housed in the Department of Geological Sciences, on 200 ppb Sr solutions. $^{87}Sr/^{86}Sr$ data presented are referenced to bracketing analyses of the international strontium isotope standard SRM987, using an $^{87}Sr/^{86}Sr$ value for this standard of 0.710255. Isobaric interference of ^{87}Rb on ^{87}Sr was corrected using the measured signal for ^{85}Rb and the natural $^{85}Rb/^{87}Rb$ ratio, while the effect of instrumental mass fractionation was corrected using the exponential law and an $^{86}Sr/^{88}Sr$ value of 0.1194. Repeat analyses of an in-house carbonate reference material NM95 as unknown during processing of samples for this study yielded an $^{87}Sr/^{86}Sr$ result (0.708912 ± 0.000037; n = 17), in agreement with long-term data for this material (0.708911 ± 0.000040 2σ; n = 414; over >8 years). Total procedural strontium blanks were typically better than ~250 pg and therefore negligible.

For statistical analysis, we tested for multimodality in the entire sample using a Hartigan's Dip test for unimodality (with values less than .05 indicating significant bimodality), and then the boot.com and normalmixEM function of the mixtools package in R[3] to search and fit different numbers of mixture components (up to five), using 1000 bootstrap realizations of the likelihood ratio statistic for the iterative

procedure. Comparison of $^{87}Sr/^{86}Sr$ values between sites was done with Wilcoxon–Mann–Whitney two-sample rank test for paired comparisons with Bonferroni correction. Additionally, we computed the effect size using r value (which is the Z value from the test divided by the total number of observations). Unlike P-values, they are not affected by sample size. R values ≥0.50 are considered large[4].

Paleodietary studies in apatite and collagen. A hand-held rotary drill fitted with a 1.2 mm diameter diamond-tipped dental drill bit was used to collect approximately 10 mg of enamel powder. The tooth was drilled from the occlusal surface to the cervix to collect enamel representative of the entire period of crown formation in order to average out seasonal variations. The enamel was treated following established method for tooth enamel pre-treatment[5]. Samples were treated with 1.75% sodium hydroxide for 45 minutes, rinsed three times with distilled water, and then reacted with 0.1M acetic acid for 15 minutes. Finally, they were rinsed three times with distilled water, freeze-dried[6]. The bone powder was treated with 1.75% sodium hypochlorite for 3 hours, rinsed three times with distilled water, then reacted with 0.1M acetic acid for 15 minutes. Samples were rinsed again and finally freeze dried. For bone collagen analyses, each sample was mechanically cleaned and weighed to enable determination of the collagen yield. The samples were then soaked overnight at room temperature in a de-fatting solution of chloroform, methanol and water (2.0:1.0:0.8 v:v). Most lipids were removed by this solution and floated to the top of the liquid. The de-fatting process was repeated if large quantities of lipids were observed. All samples were then rinsed with distilled water. Samples were then soaked in dilute HCl (0.2M) at room temperature to remove the apatite from the bone. The acid was changed every day for between five to ten days, depending on the size of the sample and the density of the bone. This resulted in a translucent, flexible pseudomorph of (mostly) collagen of the same shape and size as the original bone fragment. Each pseudomorph was soaked in in 0.1M NaOH overnight to remove base-soluble contaminants such as humic acids and any remaining lipids[7]. Next, the samples were soaked in distilled water, changed daily until its pH remained neutral. The sample was then freeze-dried and then allowed to equilibrate with the atmosphere (overnight) before being weighed to obtain the mass of collagen extracted. This is expressed as a percentage of the initial weight of the bone sample to give collagen yield. For each sample, approximately 0.5 mg of extracted collagen was weighed into a tin cup. The samples were combusted in a Flash EA 2000 series elemental analyzer (Thermo Finnigan, Milan, Italy). The gases were passed through to a Delta Plus V IRMS (Thermo electron, Bremen, Germany), via a Conflo IV gas control unit (Thermo Finnigan, Bremen, Germany). Standards used were in-house standards Chocolate ($\delta^{13}C$ = -17.75‰; $\delta^{15}N$ = 4.31‰); New MG ($\delta^{13}C$ = -20.94‰; $\delta^{15}N$ = 4.70‰; Sucrose ($\delta^{13}C$ = -10.6‰); Valine ($\delta^{13}C$ = -26.80‰; $\delta^{15}N$ = 12.14‰). Each standard had been calibrated against international standard materials NBS 21, IAEA N1 and N2 and standards exchanged with other laboratories. The reproducibility of repeated measurements of standard materials was ≤0.2‰.

Calibration and Modeling of Radiocarbon Dates. For the histogram of locals and migrants (Fig. 3B), totals for each archaeological site are shown below the dates from the same site. For Túmulo II, all 11 individuals are local, two of which are dated. The histogram bar is widened to include the temporal range suggested by the two dates. For Potrero Las Colonias, all seven individuals are migrants and there are three dates available.

There seem to be two phases of cemetery use, so four of these individuals are arbitrarily included in the histogram bar aligned with the earlier date and three with the later date. The later bar includes locals and migrants from Túmulo III. Finally, the date from Monte de Algarrobos has a bimodal distribution and its greater probability lies after European contact (AD 1610–1680, 56%) but as part of the model its median date is *AD 1530 (1500–1670, 95% probability, A=70)*. Below we present the OxCal[8] code used to generate the KDE plots in Fig. 3 and estimate the first and last date of the migrant phase.

```
Plot()
{
 Curve("SHCal20","SHCal20.14c");
 Phase()
 {
  Sequence()
  {
   Boundary("Start Locals");
   KDE_Plot("Locals")
   {
    color="green";
    R_Date("AA-66568, Túmulo I", 977, 35);
    R_Date("AA-66565, Túmulo II", 1178, 41);
    R_Date("AA-66561, Túmulo II", 1269, 35);
    Combine("Barrio Ramos")
    {
     R_Date("AA-98708, Barrio Ramos",583,43);
     R_Date("I-16636, Barrio Ramos",470,80);
     C_Date("UCTL-308, Barrio Ramos",AD(1400),60);
    };
   };
   Boundary("End Locals");
  };
  Sequence()
  {
   Boundary("Start Migrants");
   KDE_Plot("Migrants")
   {
    color="red";
    R_Date("AA-66566, Túmulo III", 671, 40);
    R_Date("D-AMS-0033193, Uspallata Usina Sur", 772, 25);
    R_Date("D-AMS-031415, Potrero Las Colonias", 634, 28);
    R_Date("AA-66564, Potrero Las Colonias", 568, 38);
    R_Date("D-AMS-033194, Potrero Las Colonias", 682, 25);
    First("First migrants");
    Last("Last migrants");
   };
   R_Date("D-AMS-030192, Monte de Algarrobo",298,28);
   Boundary("End Migrants");
  };
 };
};
```

Supplementary figures

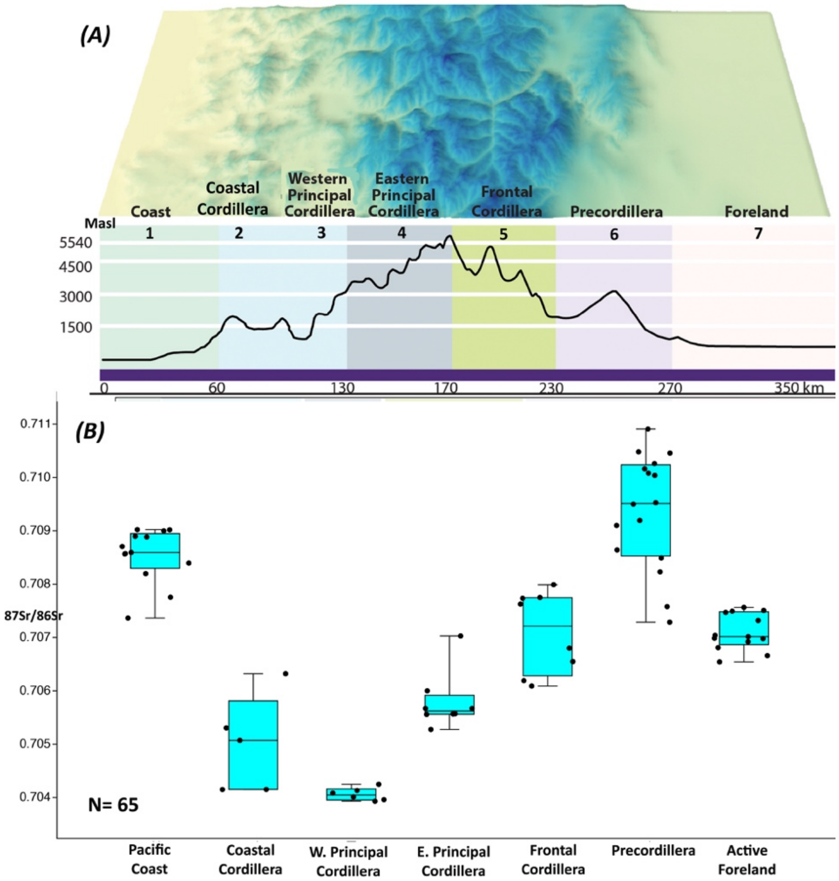

Figure S1. (a) West-east altitudinal profile of the southern Andes; **(b)** $^{87}Sr/^{86}Sr$ values for rodent samples. Map made by with Quantum GIS, Ver 3.2.3 (https://www.qgis.org), Qgis2threejs (https://github.com/minorua/Qgis2threejs) and edited with Inkscape 0.92 (https://inkscape.org).

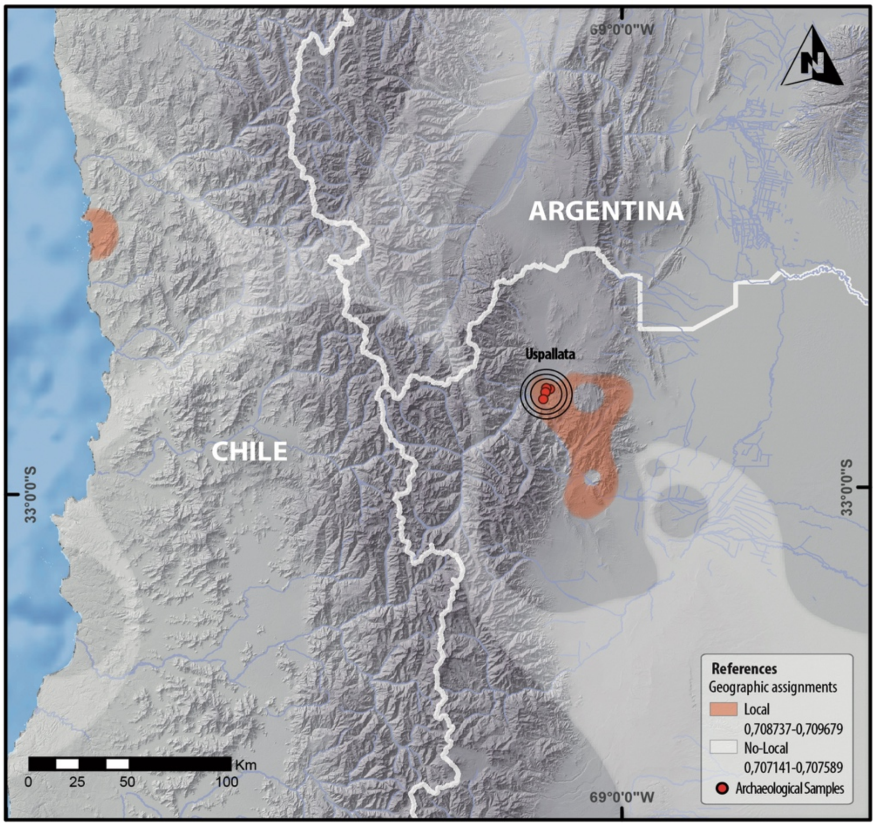

Figure S2. Areas of probable geographic residence for local and non-local individuals from the Uspallata Valley.

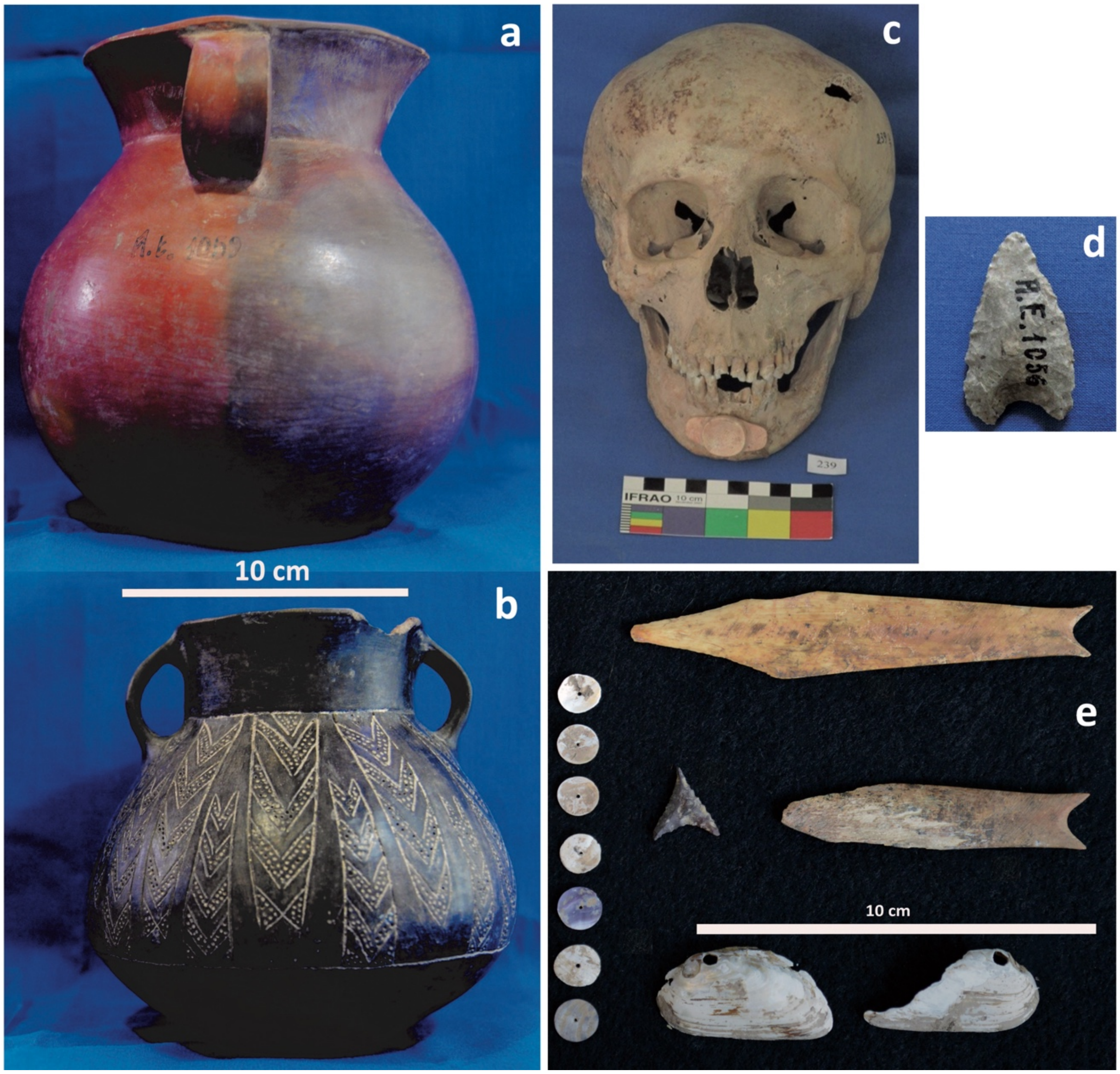

Figure S3. Grave goods from sites in the Uspallata Valley: **(a, b)** Ceramic vessels from Túmulo II, burial 245; **(c)** Cranium with lithic lip plug associated with individual 239 from Túmulo II; **(d)** Lithic projectile point associated with individual 241 from Túmulo II; **(e)** Diverse grave goods from Barrio Ramos I.

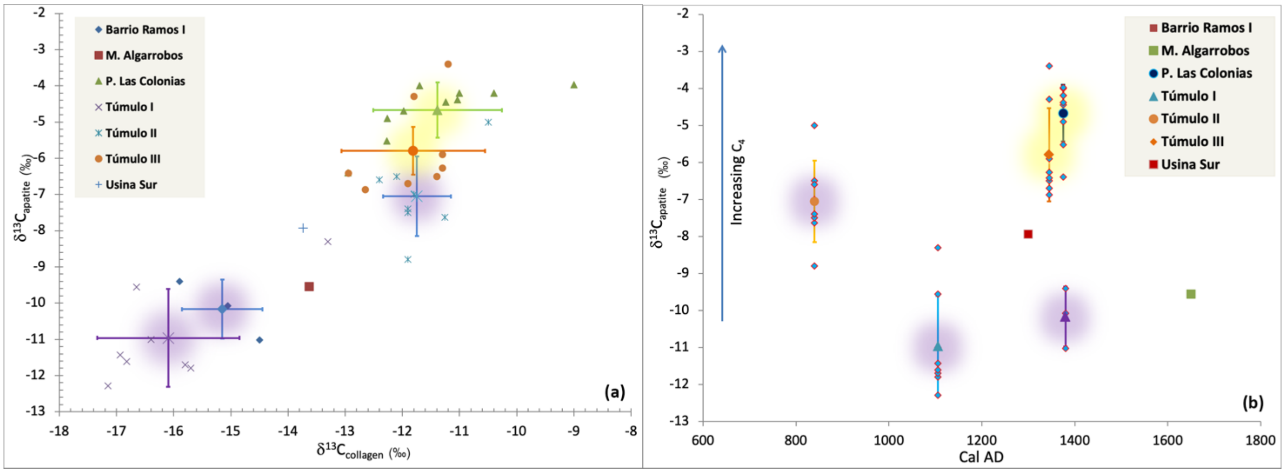

Figure S4. Paleodietary results from the Uspallata Valley: **(a)** Inter-site comparison of $\delta^{13}C_{apatite}$ and $\delta^{13}C_{collagen}$; **(b)** Temporal trends in $\delta^{13}C_{apatite}$ values (highlighted in purple: sites with locals; highlighted in yellow: sites with non-locals).

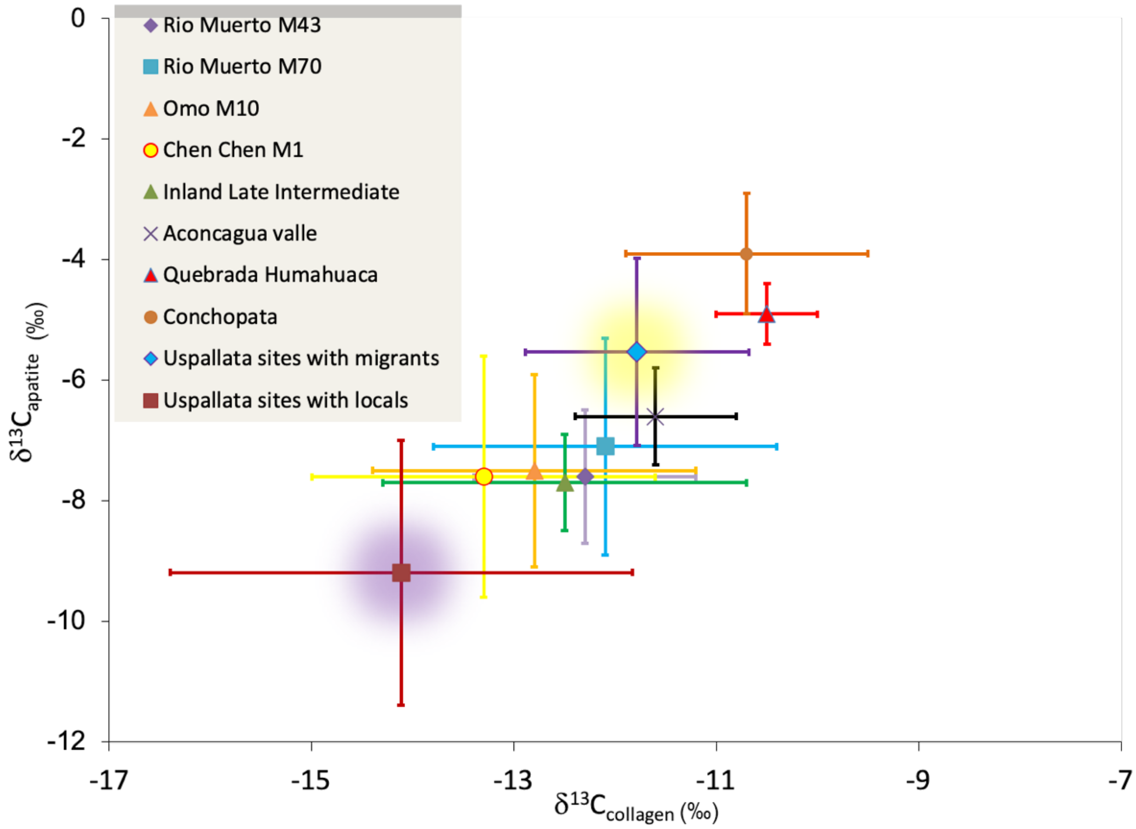

Figure S5. $\delta^{13}C_{apat.}$ and $\delta^{13}C_{coll.}$ values from key Andean sites from Peru to Central Argentina and Chile including Conchopata, Quebrada de Humahuaca, Aconcagua, Late intermediate in Chilean Central Valley, Chen Chen, Omo, Río Muerto[9–13] (highlighted in purple: sites with locals from Uspallata; highlighted in yellow: sites with non-locals from Uspallata).

Supplementary tables

Table S1. $^{87}Sr/^{86}Sr$ results for rodent samples from the southern Andes (Argentina and Chile).

N	Site	Geological unit	Latitude	Longitude	Taxon	$^{87}Sr/^{86}Sr$	Source
1	Los Vilos	Pacific Coast	262631.40	6466619.52	Rodentia	0.708395	This paper
2	Los Vilos	Pacific Coast	262631.40	6466619.52	Rodentia	0.708706	This paper
3	Los Vilos	Pacific Coast	262631.40	6466619.52	Rodentia	0.709022	This paper
4	Los Vilos	Pacific Coast	262631.40	6466619.52	Rodentia	0.708593	This paper
5	Los Vilos	Pacific Coast	262631.40	6466619.52	Rodentia	0.708885	This paper
6	Los Vilos	Pacific Coast	262631.40	6466619.52	Rodentia	0.708999	This paper
7	Los Vilos	Pacific Coast	262631.40	6466619.52	Rodentia	0.708898	This paper
8	Los Vilos	Pacific Coast	262631.40	6466619.52	Rodentia	0.709019	This paper
9	LEP-C	Pacific Coast	257578	6289821	*Myocastor coipus*	0.708572	This paper
10	LEP-C	Pacific Coast	257578	6289821	*Octodon* sp.	0.708197	This paper
11	LEP-C	Pacific Coast	257578	6289821	*Octodon* sp.	0.708566	This paper
12	Arévalo 2	Pacific Coast	259168	6282310	*Octodon* sp.	0.707365	This paper
13	Arévalo 2	Pacific Coast	259168	6282310	*Octodon* sp.	0.707754	This paper
14	RML-015	Coastal Cordillera	324598	6317284	*Octodon* sp.	0.704151	This paper
15	E-101-3	Coastal Cordillera	317900	6270000	Rodentia	0.706320	This paper
16	RML-008	Coastal Cordillera	323314	6317819	*Octodon* sp.	0.704150	This paper
17	Cerro Chena	Coastal Cordillera	337944	6279115	*Myocastor coipus*	0.705305	This paper
18	E-101-1	Coastal Cordillera	318250	6272450	*Myocastor coipus*	0.705072	This paper
19	Combarbalá	W. Principal Cordillera	309757.99	6547675.41	Rodentia	0.704083	This paper
20	Combarbalá	W. Principal Cordillera	309757.99	6547675.41	Rodentia	0.704131	This paper
21	APG	W. Principal Cordillera	325662.61	6508430.22	Rodentia	0.704249	This paper
22	Los Azules 1	W. Principal Cordillera	368949.00	6279626.00	Rodentia	0.70393	[14]
23	Manzano 1	W. Principal Cordillera	366078.00	6285315.00	Rodentia	0.70396	[14]
24	Los Queltehues	W. Principal Cordillera	387529.00	6247678.00	Rodentia	0.70401	[14]
25	Las Cuevas	E. Principal Cordillera	403034.84	6368927.96	Rodentia	0.705557	This paper
26	Las Cuevas	E. Principal Cordillera	403034.84	6368927.96	Rodentia	0.705566	This paper
27	Las Cuevas	E. Principal Cordillera	403034.84	6368927.96	Rodentia	0.705276	This paper
28	Laguna del Diamante	E. Principal Cordillera	435018.16	6215748.80	Rodentia	0.707028	This paper
29	Laguna del Diamante	E. Principal Cordillera	437689.04	6215548.34	Rodentia	0.706000	This paper
30	Las Cuevas 1	E. Principal Cordillera	403032.42	6368925.45	*Eligmodontia* sp.	0.70567	[15]
31	Las Cuevas 2	E. Principal Cordillera	403032.42	6368925.45	*Eligmodontia* sp.	0.70567	[15]

32	Aconcagua 1	E. Principal Cordillera	409520.35	6381003.54	Rodentia	0.70557	15
33	Gendarmería. L. Diamante	Frontal Cordillera	434448.00	6216494.00	Rodentia	0.70655	14
34	Quebrada de La Manga	Frontal Cordillera	470445.23	6354813.14	Rodentia	0.707626	This paper
35	Quebrada de La Manga	Frontal Cordillera	470445.23	6354813.14	Rodentia	0.707750	This paper
36	Quebrada de La Manga	Frontal Cordillera	470425.60	6354858.31	*Ctenomys* sp.	0.70799	15
37	Paramillos	Frontal Cordillera	487318.00	6406108.00	Rodentia	0.707288	This paper
38	Paramillos	Frontal Cordillera	487318.00	6406108.00	Rodentia	0.707579	This paper
39	Alero Tunduqueral	Uspallata Valley	470518.88	6400737.69	Rodentia	0.70864	14
40	Alero Tunduqueral	Uspallata Valley	470518.93	6400737.73	*Ctenomys* sp.	0.70849	15
41	Barrio Ramos	Uspallata Valley	467707.03	6393968.17	Rodentia	0.709194	This paper
42	San Ignacio	Precordillera	483332.59	6353564.45	*Galea* sp.	0.70910	15
43	San Ignacio	Precordillera	483321.59	6353588.29	Rodentia	0.708230	This paper
44	San Ignacio	Precordillera	483321.59	6353588.29	Rodentia	0.710457	This paper
45	Los Conitos	Precordillera	485344.52	6352958.45	Rodentia	0.710907	This paper
46	Los Conitos	Precordillera	485344.52	6352958.45	Rodentia	0.709528	This paper
47	Los Conitos 1	Precordillera	485354.51	6353031.54	*Galea* sp.	0.71026	15
48	Los Conitos 2	Precordillera	485354.51	6353031.54	*Phyllotis* sp.	0.70950	15
49	Agua de la Cueva	Precordillera	484649.90	6391164.90	*Phyllotis* sp.	0.71008	15
50	Agua de la Cueva	Precordillera	484649.90	6391164.90	*Ctenomys* sp.	0.71016	15
51	Agua de la Cueva	Precordillera	484649.90	6391164.90	Rodentia	0.710480	This paper
52	Agua de la Cueva	Precordillera	484649.90	6391164.90	Rodentia	0.710039	This paper
53	COINCE. San Carlos	Active foreland	501331.75	6274302.05	Rodentia	0.70732	14
54	Barrancas B61	Active foreland	523664.26	6338346.19	Rodentia	0.70666	14
55	Barrancas B61	Active foreland	523607.41	6338114.26	*Galea* sp.	0.70692	15
56	Barrancas B61	Active foreland	523607.41	6338114.26	*Galea* sp.	0.70681	15
57	Natania	Active foreland	514004.77	6357671.52	*Galea* sp.	0.70704	15
58	Divisadero	Active foreland	507761.63	6362308.71	*Ctenomys* sp.	0.70747	15
59	Natania	Active foreland	514006.13	6357668.54	Rodentia	0.706979	This paper
60	Natania	Active foreland	514006.13	6357668.54	Rodentia	0.706986	This paper
61	Lavalle	Active foreland	637003.00	6425141.00	Rodentia	0.707564	This paper
62	Lavalle	Active foreland	637003.00	6425141.00	Rodentia	0.707494	This paper
63	Capiz Alto	Active foreland	502006.97	6274554.37	Rodentia	0.707015	This paper
64	Capiz Alto	Active foreland	502006.97	6274554.37	Rodentia	0.707507	This paper
65	Barrancas B6	Active foreland	523601.20	6338119.59	Rodentia	0.706543	This paper

Table S2. Results for radiocarbon dates, radiogenic, and stable isotopes for human remains from the Uspallata Valley.

Site	Individual	^{14}C age (BP)	Laboratory code	Sex	Age (ys)	Tissue	$^{87}Sr/^{86}Sr$	Code	C:N	$\delta^{13}C_{collagen}$	$\delta^{15}N$	$\delta^{13}C_{apat.}$
Barrio Ramos I (+)	1	583±43 (*)	AA-98708	M?	35-40	rib	0.709582	MSR-296	**3.2**	**-15.9**	**10.1**	**-9.4**
Barrio Ramos I (+)	1	583±43 (*)	AA-98708	M?	35-40	I	0.709333	-	-	-	-	-
Barrio Ramos I (#)	2	583±43	AA-98708	M	40-50	LC	0.70901	UCT-24149	3.3	-15.1	9.0	-4.4
Barrio Ramos I (#)	2	583±43	AA-98708	M	40-50	rib	0.70953	UCT-24150	3.1	-14.5	8.7	-11.0
Barrio Ramos I (+, #)	3	583±43	AA-98708	F	>40	I2	0.70865	UCT-24151	3.1	-11.6	9.0	-10.0
Barrio Ramos I (+, #)	3	583±43	AA-98708	F	>40	rib	0.70922	UCT-24152	3.1	-15.1	8.7	-10.1
Monte de Algarrobos (+)	1219	298±28 (*)	D-AMS 030192 (δ^{13} = -15)	F	adult	rib	0.70757	UCT-24163	3.1	-13.6	7.7	-9.6
Monte de Algarrobos (+)	1219	298±28 (*)	D-AMS 030192	F	adult	I^2 right	0.709128	UCT-24164	3.4	-16.8	10.1	-3.9
Usina Sur 2	1	772±25 (*)	D-AMS 033193 ($\delta^{13}C$ = -6.2)	ND	adult	rib	0.707462	UCT-24161	3.3	-13.7	9.5	-7.9
Usina Sur 2	2	772±25		ND	adult	molar	0.707504	UCT-24162	3.0	-15.1	10.4	-10.4
Túmulo I	n/n 1	977±35	AA-66568	ND	adult	1st metatarsal	0.709443	UCT-24165	3.2	-16.9	9.2	-11.4
Túmulo I	n/n 2	977±35	AA-66568	ND	young adult	1st metatarsal	0.709378	UCT-24166	3.2	-16.8	8.8	-11.6
Túmulo I	n/n 3	977±35	AA-66568	ND	adult	1st metatarsal	0.709528	UCT-24167	3.1	-16.7	9.8	-9.6
Túmulo I	n/n 4	977±35	AA-66568	ND	adult	1st metatarsal	0.709156	UCT-24168	3.2	-17.2	10.4	-12.3
Túmulo II	236			ND	Sub-adult	rib	-	UCT-24171	3.0	-11.3	14.0	-7.6
Túmulo II	237			N/D	2.5-4	rib	0.708931	UCT-24172	3.1	-13.6	5.2	-
Túmulo II (+)	238			F?	8-9	I dentine-enamel	0.709073	UCT-24169	3.2	-13.1	10.1	-4.4
Túmulo II (+)	239	1269±35 (*)	AA-66561	F	>50	rib	0.709188	USF-8304	**3.2**	**-11.9**	**8.7**	**-7.5**
Túmulo II (+)	239	1269±35 (*)	AA-66561	F	>50	I_2/M2	0.708635	-	-	-	-	-
Túmulo II	240			M	40-49	rib	0.709019	MSR-2	**3.2**	**-11.9**	**9.1**	**-7.4**
Túmulo II	240			M	40-49	I_2 right	0.708342	-	-	-	-	-
Túmulo II	241			ND	35 45	rib	0.709164	MSR-3	**3.2**	**-11.8**	**9.9**	**-7.0**
Túmulo II (+)	242			M	18-23	rib	0.709002	MSR-6	**3.2**	**-10.5**	**9.7**	**-5.0**
Túmulo II (+)	242			M	18-23	I^2 right dentine-enamel	0.708639	UCT-24173	3.9	-15.5	10.2	-5.5
Túmulo II	243	1178±41 (*)	AA-66565	F	40-49	calcaneous	0.709098	USF-8293	-	**-12.1**	**10.1**	**-6.5**
Túmulo II	243	1178±41 (*)	AA-66565	F	40-49	I dentine/M 3	0.708497	UCT-24170	3.1	-13.3	10.4	-

Túmulo II	244			F	39-45	molar	0.709026	-	-	-	-	-
Túmulo II	245			M	35-45	rib	0.709038	USF-8292	-	**-12.4**	**9.8**	**6.6**
Túmulo II	245			M	35-45	I_2 right	0.709035	-	-	-	-	-
Túmulo III	n/n 1	671±40	AA-66566	ND	adult	1st metatarsal	0.708955	UCT-24174	3.2	-11.3	9.6	-6.3
Túmulo III	n/n 2	671±40	AA-66566	ND	adult	1st metatarsal	0.70879	UCT-24175	3.1	-12.7	9.6	-6.9
Túmulo III	n/n 3	671±40	AA-66566	ND	adult	1st metatarsal	0.707267	UCT-24176	3.1	-12.9	8.3	-6.4
Potrero Las Colonias	n/n 1	568±38	AA-66564	ND	sub-adult	1st metatarsal	0.707251	UCT-24153	3.2	-9	8.9	-4.0
Potrero Las Colonias	n/n 2	634±28 (*)	D-AMS 031415 ($\delta^{13}C$ = -6.6)	ND	adult	1st metatarsal	0.70732	UCT-24154	3.3	-12.3	9.2	-4.9
Potrero Las Colonias	n/n 3	682±25	D-AMS 031415	ND	adult	1st metatarsal	0.707343	UCT-24155	3.0	-12	9.5	-4.7
Potrero Las Colonias	n/n 4	682±25	D-AMS 031415	ND	adult	1st metatarsal	0.707252	UCT-24156	3.1	-11.2	9.8	-4.5
Potrero Las Colonias	n/n 5	682±25	D-AMS 031415	ND	adult	1st metatarsal	0.707407	UCT-24158	3	-12.3	9.5	-5.5
Potrero Las Colonias	n/n 6	682±25	D-AMS 031415	ND	adult	1st metatarsal	0.707243	UCT-24159	3.3	-11	9.6	-4.4
Potrero Las Colonias	n/n 7	682±25	D-AMS 031415	ND	adult	1st metatarsal	0.707401	UCT-24160	3.3	-13	9.7	-6.4

References: (*) direct date for the individual; (+) individuals included in the morphometric analyses; (#) results originally published in[14]; values in bold from[16].

Table S3. PC1 and PC2 loadings for whole skull (the most extreme values are shaded).

Landmark	Coordinate	PC1	PC2
	x	-0.035664	-0.049145
1	y	0.0014104	0.055027
	z	0.066806	0.039034
	x	-0.053533	-0.024589
2	y	-0.012895	0.030803
	z	0.06038	0.010259
	x	0.080397	0.040884
3	y	0.11613	0.065164
	z	0.077376	-0.025755
	x	0.044639	-0.041288
4	y	0.079022	0.090812
	z	0.053155	-0.02333
	x	0.0038474	-0.14992
5	y	-0.027297	-0.047223
	z	0.015634	0.078435
	x	-0.0047364	0.025474
6	y	0.015167	-0.085404
	z	-0.12526	0.1439
	x	0.048657	0.025555
7	y	-0.013058	-0.065448
	z	0.0071797	0.064986
	x	0.057789	-0.015569
8	y	-0.015394	0.0038715
	z	0.0096364	-0.026452
	x	0.040668	-0.058609
9	y	-0.0077466	0.055199
	z	0.020025	-0.017962
	x	0.1103	-0.078244
10	y	0.015228	0.0043555
	z	-0.069613	0.051146
	x	0.041363	-0.057772
11	y	0.028889	0.025535
	z	-0.015018	0.042873
	x	0.084648	-0.022087
12	y	-0.10011	-0.013661
	z	0.10799	0.085194
	x	0.072847	0.026141
13	y	0.14874	0.023368
	z	0.087229	-0.054704
	x	0.079897	-0.087291
14	y	0.11756	-0.039621
	z	0.065943	0.014818
	x	0.14845	0.12624
15	y	-0.073007	0.078459
	z	0.047412	-0.0056561
	x	-0.02341	0.0085464

16	y	0.07784	0.023487
	z	0.061937	-0.019453
	x	-0.079457	0.064081
17	y	0.015855	0.01649
	z	0.071026	-0.049339
	x	0.035222	0.0052857
	y	0.010702	-0.023308
18	z	-0.025484	-0.035342
	x	0.0087178	-0.0087723
19	y	-0.016417	0.027875
	z	0.007952	-0.025906
	x	0.043595	0.015824
20	y	-0.0094498	0.045366
	z	-0.030195	-0.045008
	x	0.00334	-0.0078751
21	y	0.02745	0.064117
	z	-0.020476	-0.029634
	x	0.00073305	-0.0029383
22	y	0.069683	0.057217
	z	-0.040431	-0.016883
	x	0.0084085	0.022319
23	y	0.11823	0.082831
	z	-0.051987	-0.029904
	x	-0.034882	0.070222
24	y	0.13451	0.039725
	z	-0.064977	0.030603
	x	-0.026174	0.038363
25	y	0.11017	0.055108
	z	-0.076153	-0.021606
	x	0.021581	0.024514
26	y	-0.0027559	0.00082094
	z	0.0092535	0.046524
	x	0.017947	-0.027669
27	y	-0.023961	0.0030416
	z	0.05218	0.032018
	x	-0.029843	-0.0049696
28	y	0.0014594	-0.027052
	z	0.019112	0.050171
	x	-0.086865	0.065254
29	y	0.019545	-0.014557
	z	-0.010038	-0.014926
	x	-0.076038	0.082586
	y	0.041258	0.028113
30	z	-0.042449	0.0061943
	x	0.047036	-0.022655
31	y	-0.073708	-0.025951
	z	0.0096186	0.01442
	x	0.093878	-0.017874

32	y	-0.13324	-0.030055
	z	-0.024939	-0.002912
33	x	-0.0042941	0.12519
	y	-0.051763	-0.005315
	z	0.04574	-0.1224
34	x	-0.034731	0.19669
	y	-0.1292	0.054885
	z	0.0094562	-0.064187
35	x	0.15453	**0.31543**
	y	-0.14283	0.12175
	z	-0.074071	0.009805
36	x	0.14588	0.034926
	y	-0.14581	0.086035
	z	-0.017646	0.046188
37	x	0.076183	0.079404
	y	-0.024949	0.038402
	z	-0.064337	0.11635
38	x	0.043668	-0.092774
	y	-0.042661	-0.10511
	z	0.035058	0.033474
39	x	0.085296	-0.056556
	y	0.12128	-0.16684
	z	0.052291	-0.042391
40	x	-0.044994	0.029677
	y	0.17699	**-0.27245**
	z	0.10798	-0.15026
41	x	-0.092614	0.061987
	y	-0.073648	-0.05838
	z	0.025412	0.00050418
42	x	-0.17795	0.038296
	y	-0.1189	-0.027309
	z	0.08184	-0.0042689
43	x	**-0.31209**	-0.14231
	y	-0.16521	0.066165
	z	0.068454	0.11671
44	x	**-0.22504**	-0.12479
	y	-0.16206	0.031334
	z	-0.10363	-0.016102
	x	0.082664	-0.043039
	y	-0.10605	-0.03606
46	z	-0.095968	-0.029314
47	x	0.01969	-0.022039
	y	-0.07001	-0.078873
	z	-0.063798	-0.018524
48	x	-0.063683	0.020469
	y	0.0063923	-0.01825
	z	-0.0041429	0.021154
	x	-0.060862	0.0047895

49	y	0.013365	-0.045277
	z	0.011134	-0.031317
50	x	0.0058561	-0.10718
	y	-0.044644	0.010765
	z	-0.026043	0.0029138
51	x	-0.057236	-0.11461
	y	0.10623	**-0.35034**
	z	0.014187	0.0060769
52	x	-0.11695	0.040807
	y	0.045987	0.071428
	z	-0.11811	-0.34436
53	x	-0.1031	-0.076264
	y	0.056252	0.0033049
	z	-0.10961	0.023438
54	x	-0.037575	-0.039255
	y	0.013325	0.053338
	z	-0.039297	0.10445
55	x	-0.060794	-0.018541
	y	0.079799	0.083166
	z	0.03037	0.093259
56	x	**0.1348**	-0.074323
	y	0.018302	0.039126
	z	-0.018102	-0.017011

Table S4. PC1 and PC2 loadings for the cranial base (the most extreme values are shaded).

Landmark	coordinate	PC1	PC2
45	x	**-0.396127**	**-0.378713**
	y	-0.183069	**0.243076**
	z	0.126871	**0.337828**
46	x	-0.090137	0.156308
	y	-0.272092	0.094864
	z	0.123102	-0.185095
47	x	0.056115	0.007001
	y	**0.232219**	0.003677
	z	0.045426	0.146927
48	x	-0.100196	-0.032812
	y	0.160067	-0.084511
	z	0.011179	**0.209794**
49	x	0.012947	-0.017182
	y	0.090837	-0.025645
	z	0.051792	**0.240764**
50	x	-0.166694	0.090051
	y	-0.128647	0.030677
	z	-0.042979	-0.073866
51	x	0.081999	-0.035399
	y	0.099830	0.194601
	z	0.037137	-0.297216
52	x	0.151755	-0.069026
	y	0.035220	0.011543
	z	0.191504	**-0.299680**
53	x	0.153716	0.064702
	y	-0.068218	0.180402
	z	0.173189	-0.072590
54	x	**0.200031**	0.091256
	y	-0.005105	-0.050963
	z	-0.171655	-0.060027
55	x	-0.045952	0.044622
	y	-0.013570	**-0.292846**
	z	**-0.558232**	0.051915
56	x	0.142543	0.079193
	y	0.052529	**-0.304875**
	z	0.012668	0.001245

Table S5. PC1 and PC2 loadings for the cranial vault (the most extreme values are shaded).

Landmark	Coordinate	PC1	PC2
31	x	0.129344	-0.040475
	y	-0.141023	-0.151392
	z	0.041775	-0.100871
32	x	0.090201	-0.036744
	y	**-0.273958**	**-0.285972**
	z	-0.012038	-0.054958
33	x	0.064458	-0.126720
	y	-0.162149	-0,072239
	z	-0.021321	0.110704
34	x	0.016754	-0.164596
	y	-0.111391	0.065667
	z	-0.022227	0.082295
35	x	0.075188	-0.145811
	y	0.082889	0.100642
	z	-0.021818	0.078396
36	x	0.206200	-0.121747
	y	0.111976	0.039295
	z	-0.009202	-0.019918
37	x	0.238788	0.225919
	y	0.093356	-0.070477
	z	0.040532	-0.102863
38	x	0.021302	**0.367995**
	y	0.124940	-0.051311
	z	0.042522	-0.021403
39	x	0.039077	**0.450128**
	y	0.265275	-0.172759
	z	0.019558	-0.002761
40	x	-0.055535	0.234011
	y	**0.383569**	-0.192769
	z	-0.000307	-0.015520
41	x	-0.045103	-0.065370
	y	**0.283436**	0.018503
	z	-0.043623	-0.001676
42	x	-0.044132	-0.070525
	y	0.151830	0.148426
	z	-0.065218	0.024768
	x	-0.193416	-0.136538

43	y	-0.086601	0.259291
	z	0.008935	-0.034122
44	x	-0.254314	-0.081518
	y	-0.293144	0.136926
	z	0.020190	0.067595
45	x	-0.192802	-0.174594
	y	**-0.285661**	0.072391
	z	0.014033	0.020100
46	x	-0.096010	-0.113414
	y	-0.143343	0.155778
	z	0.008209	-0.029768

Table S6. Paleodietary results for bone samples from the Uspallata Valley (including individuals without $^{87}Sr/^{86}Sr$ determinations).

Sample	Site	Individual	Element	Sex	Age	Lab Code	% coll.	%N	%C	C/N	$\delta^{15}N$	$\delta^{13}C_{coll.}$	$\delta^{13}C_{apat.}$	Source
1	Barrio Ramos I	1	bone fragment	ND	35-40	MSR296	ND	15.2	41.2	3.2	10.1	-15.9	-9.4	[16]
2	Barrio Ramos I	2	rib	M	40-50	24150	26	13.9	37.0	3.1	8.7	-14.5	-11.0	this paper
3	Barrio Ramos I	3	rib	F	>40	24152	24	15.8	41.3	3.1	8.7	-15.1	-10.1	this paper
4	M. Algarrobos	MM-1219	rib	F	adult	24163	25	15.7	41.4	3.1	7.7	-13.6	-9.6	this paper
5	P. Las Colonias	MMy-284	Mandible	F	30-45	USF8296	ND	ND	ND	ND	10.0	-11.0	-4.2	[16]
6	P. Las Colonias	MMy-293	rib	M?	>35	MSR210	ND	15.6	42.9	3.2	9.6	-10.4	-4.2	[16]
7	P. Las Colonias	Mmy-440	fibula diaphysis	ND	>35	MSR213	ND	15.4	42.5	3.2	9.5	-11.7	-4.0	[16]
8	P. Las Colonias	s/n 1	1st metatarsal	ND	subadult	24153	18	15.2	41.3	3.2	8.9	-9.0	-4.0	this paper
9	P. Las Colonias	s/n 2	1st metatarsal	ND	adult	24154	15	15.8	45.0	3.3	9.2	-12.3	-4.9	this paper
10	P. Las Colonias	s/n 3	1st metatarsal	ND	adult	24155	13	15.8	40.7	3.0	9.5	-12.0	-4.7	this paper
11	P. Las Colonias	s/n 4	1st metatarsal	ND	adult	24156	11	15.55	40.6	3.1	9.8	-11.2	-4.5	this paper
12	P. Las Colonias	s/n 5	1st metatarsal	ND	adult	24158	18	15.5	39.8	3.0	9.5	-12.3	-5.5	this paper
13	P. Las Colonias	s/n 6	1st metatarsal	ND	adult	24159	10	15.7	44.7	3.3	9.6	-11.0	-4.4	this paper
14	P. Las Colonias	s/n 7	1st metatarsal	ND	adult	24160	10	15.8	44.9	3.3	9.7	-13.0	-6.4	this paper
15	Túmulo I	MMy-229	parietal	Indet	>50	USF8294	ND	ND	ND	ND	9.7	-16.4	-11.0	[16]
16	Túmulo I	MMy-203-204b	phalange	ND	>35	MMy-203-204b	ND	13.5	37.3	3.2	10.1	-15.7	-11.8	[16]
17	Túmulo I	MM-220	rib	ND	>35	MM-220	ND	14.7	40.0	3.2	10.1	-13.3	-8.3	[16]
18	Túmulo I	MMy-203-204a	phalange	ND	>35	MMy-203-204a	ND	15.1	41.5	3.2	10.0	-15.8	-11.7	[16]
19	Túmulo I	s/n	1st metatarsal	ND	adult	24165	22	15.8	43.1	3.2	9.2	-16.9	-11.4	this paper
20	Túmulo I	s/n	1st metatarsal	ND	young adult	24166	16	15.6	42.6	3.2	8.8	-16.8	-11.6	this paper
21	Túmulo I	s/n	1st metatarsal	ND	adult	24167	25	15.9	42.8	3.1	9.8	-16.7	-9.6	this paper
22	Túmulo I	s/n	1st metatarsal	ND	adult	24168	22	15.9	43.8	3.2	10.4	-17.2	-12.3	this paper
23	Túmulo II	MM-236	rib	ND	infant	24171	16	15.0	39.1	3.0	14.0	-11.3	-7.6	this paper

24	Túmulo II	MMy-239	rib	F	>50	MSR-4	ND	15.0	41.2	3.2	8.7	-11.9	-7.5	[16]
25	Túmulo II	MM-240	rib	M?	40-49	MSR-2	ND	14.8	40.5	3.2	9.1	-11.9	-7.4	[16]
26	Túmulo II	MM-241	rib	ND	35-45	MSR-3	ND	14.6	40.6	3.2	9.9	-11.8	-7.0	[16]
27	Túmulo II	MM-242	rib	M	18-23	MSR-6	ND	14.7	40.6	3.2	9.7	-10.5	-5.0	[16]
28	Túmulo II	MMY-243	calcaneus	F	35-49	USF-8293	ND	ND	ND	ND	10.1	-12.1	-6.5	[16]
29	Túmulo II	MM-244	rib	F	35-49	USF8294	ND	ND	ND	ND	8.8	-11.9	-8.8	[16]
30	Túmulo II	MM-245	rib	M	35-45	USF-8292	ND	ND	ND	ND	9.8	-12.4	-6.6	[16]
31	Túmulo III	MMy-1097	bone fragment	ND	>35	USF 8297	ND	ND	ND	ND	7.8	-11.2	-3.4	[16]
32	Túmulo III	MM-1164	rib	ND	>35	MSR 5	ND	13.9	37.8	3.2	10.2	-11.4	-6.5	[16]
33	Túmulo III	MMy-1156-57a	5th metatarsal	ND	>35	MSR215	ND	12.9	35.6	3.2	9.4	-11.8	-4.3	[16]
34	Túmulo III	MMy-1156-57b	5th metatarsal	ND	infant	MSR216	ND	11.6	31.9	3.2	10.3	-11.3	-5.9	[16]
35	Túmulo III	MMy-1156-57c	5th metatarsal	ND	adult	MSR217	ND	15.2	41.4	3.2	9.9	-11.9	-6.7	[16]
36	Túmulo III	s/n	1st metatarsal	ND	adult	24174	17	15.7	42.4	3.1	9.6	-11.3	-6.3	this paper
37	Túmulo III	s/n	1st metatarsal	ND	adult	24175	19	15.5	41.6	3.1	9.6	-12.7	-6.9	this paper
38	Túmulo III	s/n	1st metatarsal	ND	adult	24176	21	15.7	41.0	3.0	8.3	-12.9	-6.4	this paper
39	Usina Sur	1	rib	ND	ND	24161	20	15.8	44.6	3.3	9.5	-13.7	-7.9	this paper

Table S7. Descriptive statistics of the stable isotopic results for archaeological sites with migrants and locals.

	Sites with migrants			Sites with locals		
	$\delta^{15}N$	$\delta^{13}C_{coll.}$	$\delta^{13}C_{apat.}$	$\delta^{15}N$	$\delta^{13}C_{coll.}$	$\delta^{13}C_{apat.}$
N	20	20	20	19	19	19
Mean	9.4	-11.8	-5.5	9.8	-14.1	-9.2
Standard deviation	0.7	1.1	1.6	1.2	2.3	2.2
Minimum value	7.7	-13.7	-9.6	8.7	-17.2	-12.3
Maximum value	10.3	-9.0	-3.4	14.0	-10.5	-5.0

Table S8. Area distribution of the stable isotopic values for migrants and locals.

	Sites with migrants	Sites with locals
Total area	10.293750	16.745750
Standard Ellipse Area (SEA)	3.677628	6.967541
Standard Ellipse Area Corrected (SEA_C)	3.881941	7.377397
Standard Ellipse Area Bayesian (SEA_B)	3.224	9.913

Table S9. List of Landmarks and semilandmarks (indicated in italic) registered for the present analysis.

N	Landmark name and abbreviation	Definition and Reference
1	Nasion (n)	Point of intersection between the frontonasal suture and the sagittal plane[22]
2	Nasofrontale (nf)	Point of intersection between the frontonasal suture and the upper lateral margin of the nasal[23]
3	Subespinale (ss)	Deepest inferior point in the nasal profile at the end of the nasal spine[24]
4	Alare (al)	Most lateral point in the nasal opening located at the transversal plane, measured at the margin of the nasal opening[24]
5	Dacryon (d)	Point at the medial border of the orbit at where the frontal, lacrimal and maxillary bones intercept. Located at the intersection between the lacrimomaxillary suture and the frontal bone[24]
6	Supraorbital Foramen (sf)	Medial point of the supraorbital foramen[25]
7	Orbital Superior (os)	Highest point on the edge of the left orbit, perpendicular to the major axis of the orbit and bisecting it[24]
8	Frontomalare Anterior (fma)	Most anterior point in the fronto-malar suture (Frontomalare orbitale)[22]
9	Ectoconchion (ek)	Opposite the dacryon, this point is in the intersection between the most anterior surface of the lateral edge of the orbit and a line that bisects it through its major axis (Ektoconkchion)[22]
10	*SL Orbital 1 (so1)*	Semilandmark located at the distal lower border of the orbit[24]
11	Zygoorbitale (zo)	Intersection between the orbital margin and the zygomaxillary suture. Registered at the midpoint, on the edge of the orbit between the facial surface and the orbital[24]
12	*SL Orbital 2 (so2)*	Semilandmark located at the lower medial end of the orbit[24]
13	Prosthion (pr)	Most prominent anterior point on alveolar border at the sagittal line, above the septum between the central incisors (Prosthion)[22]
14	Canine (can)	Most prominent point at the alveolar border, between the canine and the first premolar[23]
15	Ectomolare (em)	Most lateral point on the external surface of the alveolar border, between M2 and M3 ("Endomolare")[22]

16	Palatine (p)	Point of intersection between the palatal suture and the sagittal plane[25]
17	Alveolon (alv)	Point on the hard palate where a line drawn at the most posterior points of the alveolar edges crosses the sagittal plane[24]
18	Frontomalare Temporale (fmt)	Most lateral point on the fronto-zygomatic suture. Located posteriorly on the ridge that connects to the orbits[22]
19	*SL malar superior 1 (sms1)*	Located at the superior lateral border of the zygomatic bone, below the landmark Frontomalare Temporale[25]
20	*SL malar superior 2 (sms2)*	Located at the superior lateral border of the zygomatic bone, below sm1[25]
21	*SL malar superior 3 (sms3)*	Located at the superior lateral border of the zygomatic bone, below sm2[25]
22	*SL malar superior 4 (sms4)*	Located at the superior lateral border of the zygomatic bone, below sm3[25]
23	Temporomalare Superior (tms)	Point located at the external superior surface of the temporo-zygomatic suture[25]
24	Temporomalare Inferior (tmi)	Point located at the external lower surface of the temporo-zygomatic suture[25]
25	Temporomalare Inferior Internal (tmii)	Point located at the lower internal surface of the temporo-zygomatic suture[25]
26	Zygomaxillare Anterior (zma)	Point located inferiorly at the intersection of the zygomaxilare suture and the attachment place of the masseter muscle on the external side[25]
27	Zygomaxillare Posterior (zmp)	Point located inferiorly at the intersection of the zygomaxilare suture and the attachment place of the masseter muscle on the internal side[25]
28	*SL malar inferior 1 (smi1)*	Semilandmark located on the lower edge of the malar bone, adjacent to the zma[25]
29	*SL malar inferior 2 (smi2)*	Semilandmark located on the lower edge of the malar bone, between zma and smi3[25]
30	*SL malar inferior 3 (smi3)*	Semilandmark located on the lower edge of the malar bone, after smi2[25]
31	Glabella (g)	Most anterior point of the frontal bone, located on the sagittal plane, between the supraorbital edges, usually above the frontonasal suture (Supraorbitale)[22]
32	Frontex (f)	Point above the glabella, located at the sagittal plane, over the middle line where the frontal bone becomes more concave (Ophryon)[22]
33	*SL frontal 1 (sf1)*	Point located posteriorly to the frontex on the frontal bone[25]
34	*SL frontal 2 (sf2)*	Point located posteriorly to the sf1 on the frontal bone[25]
35	*SL frontal 3 (sf3)*	Point located between to the sf1 and bregma on the frontal bone[25]
36	Bregma (b)	Point located at the intersection between the Coronal and Sagittal sutures[22]
37	*SL parietal 1 (sp1)*	Point located posteriorly to the bregma on the sagittal suture[25]
38	*SL parietal 2 (sp2)*	Point located posteriorly to the sp1 on the sagittal suture[25]
39	*SL parietal 3 (sp3)*	Point located posteriorly to the sp2 on the sagittal suture[25]
40	*SL parietal 4 (sp4)*	Point located posteriorly to the sp3 on the sagittal suture[25]
41	Lambda (l)	Point located at the intersection between the sagittal and the lambdoid sutures[22]
42	*SL occipital 1 (soc1)*	Point located posteriorly to the lambda on the occipital bone[25]
43	*SL occipital 2 (soc2)*	Point located posteriorly the soc1 on the occipital bone[25]
44	Opisthocranion (op)	When the skull is positioned on the Frankfurt plane, most posterior point protruding from the occipital bone on the sagittal plane[22]
45	Asterion (as)	Point located at the intersection of the temporal, parietal and occipital bones[22,26]
46	Opisthion (o)	Point of intersection between the posterior edge of the foramen magnum intersects and the sagittal line (Opisthion)[22]
47	Basion (ba)	Point located at the intersection of the anterior edge of the foramen magnum and the sagittal line[22]
48	Spheno-occipital (so)	Point located at the intersection between the sphenooccipital suture and the sagittal line (Sphenobasion)[22]
49	Spheno-occipital lateral (sol)	Most lateral point located anteriorly in the spheno-occipital suture[25]
50	Temporo-occipital (to)	Most anterior point in the temporal suture located at the skull base[25]
51	Temporo-sphenoid (ts)	V-point of the spheno-temporal suture[25]
52	Temporomandibulare (™)	Point located at the posterolateral or distal border of the glenoid fossa[25]
53	Auriculare (au)	Highest point on the helix of the auricle, at the posterior ridge of the zygomatic bone at the central upper border of the auditory meatus ("Auriculare")[22]
54	Anterior Mastoid (am)	Anteriorly located at the intersection between the lower edge of the auditory meatus and the mastoid bone[25]
55	Inferior Mastoid (im)	Lowest point of the mastoid bone (Mastoideale)[22]
56	Posterior Mastoid (pm)	Most posterior point in the mastoid bone at the intersection with a projected line to the anterior mastoid[25]

Table S10. List of studied specimens for 3D geometric morphometrics.

Code	Site	$^{87}Sr/^{86}Sr$ signature	Sex determination	Age estimation	95% cal range (AD)
229	Túmulo I	Local	Indetermined	>50	1020–1190
230	Túmulo I	Local	Masculine	>50	1020–1190
237	Túmulo II	Local	Indetermined	2.5-4	770–1000
240	Túmulo II	Local	Masculine	40-49	770–1000
243	Túmulo II	Local	Femenine	35-49	770–1000
245	Túmulo II	Local	Masculine	35-45	770–1000
238	Túmulo II	Local	Femenine	9-11	770–1000
239	Túmulo II	Local	Femenine	>50	680–890
242	Túmulo II	Local	Masculine	18-23	770–1000
1	Barrio Ramos I	Local	Masculine	35-40	1310–1450
2	Barrio Ramos I	Local	Masculine	40-50	1310–1450
3	Barrio Ramos I	Local	Femenine	>40	1310–1450
1138	Túmulo III	Non-local	Indetermined	Adult	1270–1420
1219	Monte de Algarrobos	Non-local	Femenine	35-45	1500–1800
297	Potrero Las Colonias	Non-local	Femenine	Adult	1290–1430
298	Potrero Las Colonias	Non-local	Masculine	20-30	1290–1430
305	Potrero Las Colonias	Non-local	Masculine	>45	1290–1430
807	Potrero Las Colonias	Non-local	Femenine	30-45	1290–1430
808	Potrero Las Colonias	Non-local	Femenine	10-12	1290–1430
810	Potrero Las Colonias	Non-local	Masculine	Adult	1290–1430
1203	Potrero Las Colonias	Non-local	Masculine	30-35	1290–1430

Supplementary references

1. Harris, C. The oxygen isotope composition of Karoo and Etendeka picrites: high $\delta^{18}O$ mantle or crustal contamination? *Contrib Miner. Pet.* **170**, 8 (2015).
2. Pin, C. Concominant Separation of strontium and samarium–neodymium for isotopic analysis in silicate samples, based on specific extraction chromatography. *Anal Chim Acta* **298**, 209–217 (1994).
3. R Core Team. (2013).
4. Cohen, J. *Statistical power analysis for the behavioral sciences*. (Erlbaum, 1988).
5. Lee-Thorp, J. *et al.* Exploring problems and opportunities offered by down-scaling sample sizes for carbon isotope analyses of fossils. *Bull Soc Geol Fr* **168**, 767–773 (1997).
6. Luyt, J., Hare, V. J. & Sealy, J. The relationship of ungulate $\delta^{13}C$ and environment in the temperate biome of southern Africa, and its palaeoclimatic application. *Palaeogeogr. Palaeoclimatol. Palaeoecol.* **514**, 282–291 (2019).
7. Ambrose, S. H. Preparation and characterization of bone and tooth collagen for isotopic analysis. *J. Archaeol. Sci.* **17**, 431–451 (1990).
8. Bronk Ramsey, C. Bayesian Analysis of Radiocarbon Dates. *Radiocarbon* **51**, 337–360 (2009).
9. Finucane, B. C. Maize and Sociopolitical Complexity in the Ayacucho Valley, Peru. *Curr. Anthropol.* **50**, 535–545 (2009).
10. Falabella, F., Planella, M. T. & Tykot, R. H. El Maíz (*Zea mays*) en el Mundo Prehispánico de

Chile Central. *Lat. Am. Antiq.* **19**, 25–46 (2008).
11. Alfonso-Durruty, M., Troncoso, A., Larach, P., Becker, C. & Misarti, N. Maize (*Zea mays*) consumption in the southern andes (30°-31° S. Lat): Stable isotope evidence (2000 BCE-1540 CE). *Am. J. Phys. Anthropol.* **164**, 148–162 (2017).
12. Somerville, A. D. *et al.* Diet and gender in the Tiwanaku colonies: Stable isotope analysis of human bone collagen and apatite from Moquegua, Peru. *Am. J. Phys. Anthropol.* **158**, 408–422 (2015).
13. Killian Galván, V., Seldes, V. & Nielsen, A. E. Inferencia paleodietaria en el sitio arqueológico los amarillos (Quebrada de Humahuaca, Jujuy, Argentina). *Relac. Soc. Argent. Antropol.* **XLI**, 79–99 (2016).
14. Barberena, R. *et al.* Scale of human mobility in the southern Andes (Argentina and Chile): A new framework based on strontium isotopes. *Am. J. Phys. Anthropol.* **164**, 305–320 (2017).
15. Barberena, R. *et al.* Bioavailable Strontium in the Southern Andes (Argentina and Chile): A Tool for Tracking Human and Animal Movement. *Environ. Archaeol.* 1–13 (2019) doi:10.1080/14614103.2019.1689894.
16. Gil, A. F. *et al.* Isotopic evidence on human bone for declining maize consumption during the little ice age in central western Argentina. *J. Archaeol. Sci.* **49**, 213–227 (2014).
17. Martin, R. & Saller, K. *Lehrbuch der Anthropologie*. (Fisher, 1957).
18. Gonzalez, P. N., Perez, S. I. & Bernal, V. Ontogenetic allometry and cranial shape diversification among human populations from south America. *Anat. Rec.* **294**, (2011).
19. Howells, W. W. Cranial variation in man. A study by multivariate analysis of patterns of difference among recent human populations. *Pap. Peabody Mus. Archaeol.* **67**, 1–259 (1973).
20. Menéndez, L. P. *Diversificación Morfológica Craneofacial y Diversidad en la Dieta. El Caso de la Región Centro-Oeste de Argentina durante el Holoceno tardío*. vol. S2743 (Archaeopress, 2015).
21. Bookstein, F. L. Landmark methods for forms without landmarks: morphometrics of group differences in outline shape. *Med Image Anal* **1**, 225–243 (1997).
